# Risk factors for adverse outcomes during mechanical ventilation of 1152 COVID-19 patients: a multicenter machine learning study with highly granular data from the Dutch Data Warehouse

**DOI:** 10.1186/s40635-021-00397-5

**Published:** 2021-06-28

**Authors:** Lucas M. Fleuren, Michele Tonutti, Daan P. de Bruin, Robbert C. A. Lalisang, Tariq A. Dam, Diederik Gommers, Olaf L. Cremer, Rob J. Bosman, Sebastiaan J. J. Vonk, Mattia Fornasa, Tomas Machado, Nardo J. M. van der Meer, Sander Rigter, Evert-Jan Wils, Tim Frenzel, Dave A. Dongelmans, Remko de Jong, Marco Peters, Marlijn J. A. Kamps, Dharmanand Ramnarain, Ralph Nowitzky, Fleur G. C. A. Nooteboom, Wouter de Ruijter, Louise C. Urlings-Strop, Ellen G. M. Smit, D. Jannet Mehagnoul-Schipper, Tom Dormans, Cornelis P. C. de Jager, Stefaan H. A. Hendriks, Evelien Oostdijk, Auke C. Reidinga, Barbara Festen-Spanjer, Gert Brunnekreef, Alexander D. Cornet, Walter van den Tempel, Age D. Boelens, Peter Koetsier, Judith Lens, Sefanja Achterberg, Harald J. Faber, A. Karakus, Menno Beukema, Robert Entjes, Paul de Jong, Taco Houwert, Hidde Hovenkamp, Roberto Noorduijn Londono, Davide Quintarelli, Martijn G. Scholtemeijer, Aletta A. de Beer, Giovanni Cinà, Martijn Beudel, Nicolet F. de Keizer, Mark Hoogendoorn, Armand R. J. Girbes, Willem E. Herter, Paul W. G. Elbers, Patrick J. Thoral, Dutch ICU Data Sharing Against COVID-19 Collaborators, Thijs C. D. Rettig, M. C. Reuland, Laura van Manen, Leon Montenij, Jasper van Bommel, Roy van den Berg, Ellen van Geest, Anisa Hana, W. G. Boersma, B. van den Bogaard, Peter Pickkers, Pim van der Heiden, Claudia C. W. van Gemeren, Arend Jan Meinders, Martha de Bruin, Emma Rademaker, Frits H. M. van Osch, Martijn de Kruif, Nicolas Schroten, Klaas Sierk Arnold, J. W. Fijen, Jacomar J. M. van Koesveld, Koen S. Simons, Joost Labout, Bart van de Gaauw, Michael Kuiper, Albertus Beishuizen, Dennis Geutjes, Johan Lutisan, Bart P. X. Grady, Remko van den Akker, Bram Simons, A. A. Rijkeboer, Sesmu Arbous, Marcel Aries, Niels C. Gritters van den Oever, Martijn van Tellingen, Annemieke Dijkstra, Rutger van Raalte, Luca Roggeveen, Fuda van Diggelen, Ali el Hassouni, David Romero Guzman, Sandjai Bhulai, Dagmar Ouweneel, Ronald Driessen, Jan Peppink, H. J. de Grooth, G. J. Zijlstra, A. J. van Tienhoven, Evelien van der Heiden, Jan Jaap Spijkstra, Hans van der Spoel, Angelique de Man, Thomas Klausch, Heder de Vries, Michael de Neree tot Babberich, Olivier Thijssens, Lot Wagemakers, Hilde G. A. van der Pol, Tom Hendriks, Julie Berend, Virginia Ceni Silva, Bob Kullberg, Leo Heunks, Nicole Juffermans, Arjan Slooter

**Affiliations:** 1 Department of Intensive Care Medicine, Amsterdam UMC, Amsterdam, The Netherlands; 2 Pacmed, Amsterdam, The Netherlands; 3 Department of Intensive Care, Erasmus Medical Center, Rotterdam, The Netherlands; 4 Intensive Care, UMC Utrecht, Utrecht, The Netherlands; 5 ICU, OLVG, Amsterdam, The Netherlands; 6 Intensive Care, Amphia Ziekenhuis, Breda en Oosterhout, The Netherlands; 7 Department of Anesthesiology and Intensive Care, St. Antonius Hospital, Nieuwegein, The Netherlands; 8 Department of Intensive Care, Franciscus Gasthuis & Vlietland, Rotterdam, The Netherlands; 9 Department of Intensive Care Medicine, Radboud University Medical Center, Nijmegen, The Netherlands; 10 Intensive Care, Bovenij Ziekenhuis, Amsterdam, The Netherlands; 11 Intensive Care, Canisius Wilhelmina Ziekenhuis, Nijmegen, The Netherlands; 12 Intensive Care, Catharina Ziekenhuis Eindhoven, Eindhoven, The Netherlands; 13 Intensive Care, ETZ Tilburg, Tilburg, The Netherlands; 14 Intensive Care, Haga Ziekenhuis, Den Haag, The Netherlands; 15 Intensive Care, Laurentius Ziekenhuis, Roermond, The Netherlands; 16 Intensive Care, Noordwest Ziekenhuisgroep, Alkmaar, Netherlands; 17 Intensive Care, Reinier de Graaf Gasthuis, Delft, The Netherlands; 18 Intensive Care, Spaarne Gasthuis, Haarlem en Hoofddorp, The Netherlands; 19 Intensive Care, VieCuri Medisch Centrum, Venlo, The Netherlands; 20 Intensive Care, Zuyderland MC, Heerlen, The Netherlands; 21 Department of Intensive Care, Jeroen Bosch Ziekenhuis, Den Bosch, The Netherlands; 22 Intensive Care, Albert Schweitzerziekenhuis, Dordrecht, The Netherlands; 23 ICU, Maasstad Ziekenhuis Rotterdam, Rotterdam, The Netherlands; 24 ICU, SEH, BWC, Martiniziekenhuis, Groningen, The Netherlands; 25 Intensive Care, Ziekenhuis Gelderse Vallei, Ede, The Netherlands; 26 Department of Intensive Care, Ziekenhuisgroep Twente, Almelo, The Netherlands; 27 Department of Intensive Care, Medisch Spectrum Twente, Enschede, The Netherlands; 28 Department of Intensive Care, Ikazia Ziekenhuis Rotterdam, Rotterdam, The Netherlands; 29 Anesthesiology, Antonius Ziekenhuis Sneek, Sneek, The Netherlands; 30 Intensive Care, Medisch Centrum Leeuwarden, Leeuwarden, The Netherlands; 31 ICU, IJsselland Ziekenhuis, Capelle aan den IJssel, The Netherlands; 32 ICU, Haaglanden Medisch Centrum, Den Haag, The Netherlands; 33 ICU, WZA, Assen, The Netherlands; 34 Department of Intensive Care, Diakonessenhuis Hospital, Utrecht, The Netherlands; 35 Department of Intensive Care, Streekziekenhuis Koningin Beatrix, Winterswijk, The Netherlands; 36 Department of Intensive Care, Admiraal De Ruyter Ziekenhuis, Goes, The Netherlands; 37 Department of Anesthesia and Intensive Care, Slingeland Ziekenhuis, Doetinchem, The Netherlands; 38 Department of Neurology, Amsterdam UMC, Universiteit Van Amsterdam, Amsterdam, The Netherlands; 39 Department of Clinical Informatics, Amsterdam UMC, Amsterdam, The Netherlands; 40 Quantitative Data Analytics Group, Department of Computer Science, Faculty of Science, VU University, Amsterdam, The Netherlands

**Keywords:** COVID-19, Mortality prediction, Risk factors, Machine learning

## Abstract

**Background:**

The identification of risk factors for adverse outcomes and prolonged intensive care unit (ICU) stay in COVID-19 patients is essential for prognostication, determining treatment intensity, and resource allocation. Previous studies have determined risk factors on admission only, and included a limited number of predictors. Therefore, using data from the highly granular and multicenter Dutch Data Warehouse, we developed machine learning models to identify risk factors for ICU mortality, ventilator-free days and ICU-free days during the course of invasive mechanical ventilation (IMV) in COVID-19 patients.

**Methods:**

The DDW is a growing electronic health record database of critically ill COVID-19 patients in the Netherlands. All adult ICU patients on IMV were eligible for inclusion. Transfers, patients admitted for less than 24 h, and patients still admitted at time of data extraction were excluded. Predictors were selected based on the literature, and included medication dosage and fluid balance. Multiple algorithms were trained and validated on up to three sets of observations per patient on day 1, 7, and 14 using fivefold nested cross-validation, keeping observations from an individual patient in the same split.

**Results:**

A total of 1152 patients were included in the model. XGBoost models performed best for all outcomes and were used to calculate predictor importance. Using Shapley additive explanations (SHAP), age was the most important demographic risk factor for the outcomes upon start of IMV and throughout its course. The relative probability of death across age values is visualized in Partial Dependence Plots (PDPs), with an increase starting at 54 years. Besides age, acidaemia, low *P*/*F*-ratios and high driving pressures demonstrated a higher probability of death. The PDP for driving pressure showed a relative probability increase starting at 12 cmH_2_O.

**Conclusion:**

Age is the most important demographic risk factor of ICU mortality, ICU-free days and ventilator-free days throughout the course of invasive mechanical ventilation in critically ill COVID-19 patients. pH, *P*/*F* ratio, and driving pressure should be monitored closely over the course of mechanical ventilation as risk factors predictive of these outcomes.

**Supplementary Information:**

The online version contains supplementary material available at 10.1186/s40635-021-00397-5.

## Background

Since December 2019, coronavirus disease 2019 (COVID-19) has quickly spread around the world [1]. Many countries have experienced high mortality rates and overburdened intensive care units (ICUs) [2]. Although many COVID-19 registries have improved our understanding of patient characteristics upon ICU admission [3–5], much remains to be elucidated about the predictors of mortality and length of stay in critically ill COVID-19 patients. In particular, a better understanding of these predictors could aid clinicians in the prognosis of critically ill patients and may aid policy-makers and medical professionals in optimizing resource allocation. This is of pivotal importance at the time of possible ICU admission, but also throughout the entire course of ICU treatment.

Currently, multicenter and ICU-tailored predictive modeling is scarce for COVID-19 patients. Prognostication in COVID-19 has largely centered around severity of disease, ICU admission, need for mechanical ventilation, length of stay and mortality in the general hospital population [6–8]. In addition, ICU-specific models often fail to incorporate the wide variety of dedicated ICU therapies such as mechanical ventilation or high-risk medication. Furthermore, many of these models are single center and are frequently limited to risk factors at ICU admission, while COVID-19 often requires lengthy intensive care stays. Lastly, many prognostication models lacked adherence to established documentation guidelines and principles of data sharing to improve reproducibility of predictive studies [6]. Overall, we identified a gap for reproducible, multicenter predictive models in the ICU that include ICU-specific predictors over time.

In this study, we aim to identify the risk factors for intensive care mortality, ICU-free days and ventilator-free days throughout the duration of invasive mechanical ventilation (IMV), focusing on the first, 7th and 14th day after intubation. For these analyses, it is essential to capture all available data throughout ICU admission. We therefore relied on the Dutch Data Warehouse (DDW), a large, observational, multicenter collaboration uniting 66 out of 81 intensive care units in the Netherlands [9]. Our hypothesis is that ICU treatment characteristics become more important as predictors of outcome throughout the course of IMV.

## Methods

The Medical Ethics Committee at Amsterdam UMC, location Vrije Universiteit medical center (VUmc) waived the need for patient informed consent and approved an opt-out procedure for the collection of COVID-19 patient data during the COVID-19 crisis. This report follows the transparent reporting of a multivariable prediction model for individual prognosis or diagnosis (TRIPOD) guideline [10].

### Source of data

The DDW is coordinated by Amsterdam UMC and is supported by the Dutch Society for Intensive Care (NVIC) and the Foundation for National Intensive Care Evaluation (NICE). The highly granular data warehouse is continuously expanding and currently contains over 400 million data points combining electronic health record (EHR) data from 25 hospitals on 2382 critically ill COVID-19 patients throughout their ICU treatment. A more detailed description of the DDW, the data ingestion and the data preprocessing has been published previously [9]. In brief, data were extracted in the highest frequency available, routine hourly or bihourly measurements, or at least multiple measurements per day. Data were pseudonymized in the participating hospitals. Because of the variation in parameter names between hospitals, each parameter name from a hospital was mapped to a list of predefined parameter names. Data entry errors were filtered, and derived parameters were added. The continuous data validation process included checkpoints for correct mapping, source hospital verification and distribution plots of all used parameters. The resulting data are available for researchers and clinicians within ethical and legal boundaries [11].

### Patient population

All adult intensive care patients with COVID-19 on invasive mechanical ventilation from the participating hospitals were included in this study. Additional file 1: Fig. S1 outlines the patient selection process. Patients were admitted between the beginning of the crisis in March 2020 until the 23rd of January 2021. COVID-19 was defined as a positive real-time reverse transcriptase polymerase chain reaction (RT-PCR) assay for SARS-CoV-2 or a COVID-19 Reporting and Data System (CO-RADS) score and clinical suspicion with no obvious other cause of respiratory distress [12]. Patients still admitted at data extraction as well as transfers were excluded since their outcomes are unknown. Admissions lasting less than 24 h were removed because they lack sufficient data for modeling.

### Outcomes and predictors

The primary outcomes of the study were intensive care mortality, ICU-free days in 30 days and ventilator-free days in 30 days [13]. The ICU-free days describe the number of days a patient is alive and outside of the ICU in the first 30 days after prediction. Similarly, ventilator-free days describe the days patients are alive and without invasive mechanical ventilation within the first 30 days after prediction. By definition, both outcomes were set to 0 for patients who died in the ICU within the 30-day time window.

To streamline the nomenclature from the statistical and machine learning field, all independent variables, also known as features, included in the modeling are referred to as predictors. All items from the Simplified Acute Physiology Score II (SAPSII) and sequential organ failure assessment (SOFA) score were included as predictors [14, 15]. For the ventilatory predictors, all variables from the landmark paper relating driving pressure to survival by Amato et al. were included [16]. A team of 3 experienced intensive care clinicians reviewed the list and added potentially relevant predictors. These included structured comorbidity data routinely collected for the Dutch National Intensive Care Evaluation (NICE), as well as information regarding the patient positioning and ventilation characteristics. Notably, fluid balance and the total equivalent dose of vasopressors and steroids administered were included. Finally, the length of intubation for each observation, in days, was also used as a predictor. A full list of predictors, medications, comorbidities and their definitions can be found in Additional file 1: Table S1.

### Modeling

Up to three observations were constructed for each patient depending on their length of stay, averaging the available predictor values in the 24 h preceding 1 day, 7 days, and 14 days of IMV. This process is illustrated in Additional file 1: Fig. S2. ICU mortality was modeled as a classification problem with a decision tree, logistic regression and XGBoost algorithm to investigate the performance of both simple and complex linear and non-linear models. Ventilator and ICU-free days were treated as regression problems with a Lasso and Ridge linear model, as well as an XGBoost regressor. For every outcome and every algorithm, a single model was fit on data points from all days.

Overall model performance was evaluated using the area under the receiver operating characteristic (AUROC), average precision, calibration loss, and Brier score. A nested cross-validation was performed for hyperparameter optimization and to assess performance on the whole dataset. This approach first splits the data into five outer holdout sets with 20% of the data each. For each holdout set, the remaining 80% of the data were used to fit and optimize a model via fivefold cross-validation and a randomized search over a predefined range of hyperparameter values. Observations belonging to the same patient were always kept in the same set to avoid leakage of information. A graphical representation of the process is shown in Additional file 1: Fig. S3.

For each outer holdout set, data imputation, standardization and automated feature selection were performed independently on each train set and then applied to the test set. Missing data were imputed using median imputation for simplicity and predictors were standardized to have a mean of 0 and a standard deviation of 1. A Lasso regression was used for automatic feature selection [17], and its L1 regularization term was optimized together with the classifiers’ hyperparameters. The best-performing estimator from each inner cross-validation was then used to predict the performance on the corresponding holdout test set. The overall performance resulted from the average performance of all outer folds.

### Predictor importance and interpretation

In order to interpret the models, each algorithm was retrained on the whole dataset using the best hyperparameters found by the nested cross-validation. The importance of the individual predictors was gauged using the Shapley additive explanation (SHAP) framework [18]. SHAP values are state of the art in machine learning explainability and represent the marginal contribution of a predictor to the overall prediction. Interventional SHAP values were calculated separately for each observation in the dataset [19], and then grouped to find the mean predictor importance on the different days and the whole dataset. In addition, Partial Dependence Plots (PDPs) were created for each predictor [20]. PDPs show the average change in probability of the outcome for all values of a predictor, while keeping all other predictors constant. All analyses were performed in Python 3.8 (Python Software Foundation).

### Role of the funding source

The sponsors had no role in any part of the design of the study, data collection, analysis, interpretation of data, the writing of the report nor the decision to submit.

## Results

### Cohort description

A total of 1152 patients were on invasive mechanical ventilation and included in the modeling. 883 of these patients were admitted before the 1st of September 2020 during the first wave in the Netherlands, 269 patients were admitted after this date during the second wave. Compared to day 1761 patients were mechanically ventilated for more than 7 days (66%), and 383 for more than 14 days (33%). Patient demographics, lab values, and respiratory characteristics are summarized in Table 1 for the different prediction timepoints throughout the course of IMV. For the total cohort on day 1, median age was 66 years (IQR 58–72 years), the majority were male (73%), and the median body mass index (BMI) was 27.8 (IQR 25.3–31.5 kg/m^2^).

**Table 1:** Patient characteristics on day 1, day 7 and day 14 of invasive mechanical ventilation

	Day 1	Day 7	Day 14
	(*N* = 1152)	(*N* = 761)	(*N* = 383)
Male	73% (*N* = 1139)	74% (*N* = 756)	76% (*N* = 380)
Age, years	66 (58–72, *N* = 1126)	65 (58–72, *N* = 753)	66 (58–72, *N* = 381)
< 60	33%	33%	33%
60–70	35%	36%	35%
70–80	30%	29%	31%
> 80	2%	2%	2%
BMI, kg/m^2^	27.8 (25.3–31.5, *N* = 988)	28.4 (25.5–31.9, *N* = 670)	28.1 (25.6–31.7, *N* = 328)
< 25	23%	22%	23%
25–30	44%	43%	44%
30–35	21%	22%	20%
> 35	12%	13%	13%
ICU mortality	28.8% (*N* = 1152)	30.4% (*N* = 761)	32.4% (*N* = 383)
ICU-free days	7 (0–21, *N* = 1152)	6 (0–21, *N* = 761)	3 (0–16, *N* = 383)
Ventilator-free days	15 (0–23, *N* = 1152)	16 (0–24, *N* = 761)	18 (0–25, *N* = 383)
*Laboratory*			
CRP, mg/L	18 (104–267, *N* = 999)	171 (82–266, *N* = 718)	126 (60–196, *N* = 364)
Creatinine, micromol/L	83 (65–119, *N* = 1068)	89 (64–148, *N* = 732)	93 (61–156, *N* = 364)
D-dimer, ng/mL	1522 (893–3423, *N* = 354)	2600 (1509–4976, *N* = 417)	3120 (1900–4770, *N* = 241)
Lactate, mmol/L	1.2 (1.0–1.6, *N* = 1005)	1.2 (0.9–1.6, *N* = 692)	1.2 (0.9–1.4, *N* = 348)
Leukocytes, 10^9^/L	9.7 (7.2–12.8, *N* = 1071)	10.5 (7.9–13.9, *N* = 743)	12.2 (9.7–15.5, *N* = 373)
pH	7.37 (7.32–7.41, *N* = 1105)	7.41 (7.35–7.46, *N* = 742)	7.4 (7.33–7.46, *N* = 369)
Thrombocytes, 10^9^/L	251 (189–325, *N* = 1093)	309 (225–397, *N* = 748)	383 (281–507, *N* = 376)
*Respiratory parameters*			
Respiratory rate, /min	22 (20–26, *N* = 846)	24 (20–28, *N* = 618)	25 (22–28, *N* = 324)
FiO_2_, %	45 (40–55, *N* = 1124)	46 (40–58, *N* = 754)	45 (36–60, *N* = 382)
PEEP, cmH_2_O	12 (10–14, *N* = 1121)	12 (10–14, *N* = 758)	10 (8–13, *N* = 383)
Pressure control:			
Set pressure, cmH_2_O	12 (10–15, *N* = 816)	12 (8–16, *N* = 655)	12 (8–16, *N* = 346)
Volume control:			
Plat pressure, cmH_2_O	24 (21–27, *N* = 528)	25 (22–29, *N* = 481)	25 (21–29, *N* = 276)
Tidal volume, mL/kg PBW	6.6 (6.1–7.6, *N* = 1085)	6.8 (6.1–7.8, *N* = 729)	6.9 (6.1–8.0, *N* = 362)
Static compliance^a^*, *ml/cmH_2_O	38 (30–52, *N* = 911)	37 (28–57, *N* = 671)	37 (26–60, *N* = 341)
Driving pressure, cmH_2_O	12 (9–14, *N* = 937)	12 (8–16, *N* = 684)	13 (9–16, *N* = 352)
*P*/*F*-ratio^b^	167 (130–210, *N* = 1110)	152 (122–193, *N* = 756)	161 (120–203, *N* = 380)
Ventilatory ratio^c^	1.7 (1.3–2.2, *N* = 1046)	2.1 (1.7–2.7, *N* = 718)	2.3 (1.8–2.9, *N* = 357)

Patient demographics, lab values and respiratory parameters are shown. All values represent the median with an interquartile range unless otherwise specified. The number of observations is included. Respiratory parameters and gas exchange indices were shown for patients in a controlled mode only

Patient demographics did not change substantially between the different days on IMV

*PC* pressure control, *PWB* predicted body weight, *plat pressure* plateau pressure, *FiO*_*2*_ fraction of inspired oxygen, *PEEP* positive end expiratory pressure, *CRP* C-reactive protein

^a^The recorded static respiratory system compliance or the tidal volume/(plateau pressure—PEEP)

^b^Gradient between PaO_2_ and FiO_2_

^c^Minute volume * PCO_2_/(predicted body weight * 100 * 37.5)

Interestingly, mortality during ICU admission occurred in 28.8% of patients that survived at least 24 h on IMV and only slightly increased throughout the course of mechanical ventilation; 32.4% of patients that survived up until day 14 on IMV still died afterwards. Median ventilator-free days on day 30 were 15 days (IQR 0–23) for the entire cohort and median ICU-free days were 7 days (IQR 0–21). The median C-reactive protein (CRP) decreased throughout these time points, whereas the leukocytes increased. Of note, the ventilatory ratio (minute volume * PCO_2_/(predicted body weight * 100 * 37.5)) and the D-dimer increased with longer ICU admission.

### Model results

The overall model results and the results on the different days of IMV are presented in Table 2 for ICU mortality and ICU and ventilator-free days after 30 days. Additional performance metrics can be found in Additional file 1: Table S2. The XGBoost algorithm yielded the highest performance for each of the outcomes, as well as an increase in performance later into the IMV course. Given the performance and ability of the XGBoost algorithm to encode the interaction between possibly non-linear predictors, the predictor importance was produced with this model.

**Table 2:** Model performance for the different outcomes and day of IMV

	Overall	Day 1	Day 7	Day 14
*ICU mortality (AUROC ± 95% confidence interval)*				
Decision tree	0.695 ± 0.027	0.668 ± 0.042	0.718 ± 0.013	0.739 ± 0.051
Logistic regression	0.744 ± 0.023	0.710 ± 0.035	0.766 ± 0.024	0.782 ± 0.028
XGBoost	0.774 ± 0.023	0.732 ± 0.04	0.806 ± 0.025	0.817 ± 0.013
*ICU-free days (R2 ± 95% confidence interval)*				
Lasso	0.118 ± 0.009	0.086 ± 0.024	0.147 ± 0.016	0.067 ± 0.100
Ridge	0.179 ± 0.050	0.140 ± 0.065	0.196 ± 0.071	0.229 ± 0.081
XGBoost	0.212 ± 0.028	0.148 ± 0.029	0.267 ± 0.090	0.263 ± 0.077
*Ventilator-free days (R2 ± 95% confidence interval)*				
Lasso	0.169 ± 0.015	0.112 ± 0.012	0.209 ± 0.050	0.231 ± 0.024
Ridge	0.217 ± 0.038	0.147 ± 0.018	0.263 ± 0.108	0.303 ± 0.039
XGBoost	0.250 ± 0.033	0.160 ± 0.019	0.319 ± 0.080	0.352 ± 0.038

Model performance is shown for ICU mortality, ventilator-free days at day 30, and ICU-free days at day 30 across the days of IMV

*AUROC* area under the receiver operating characteristic

**Fig. 1:**
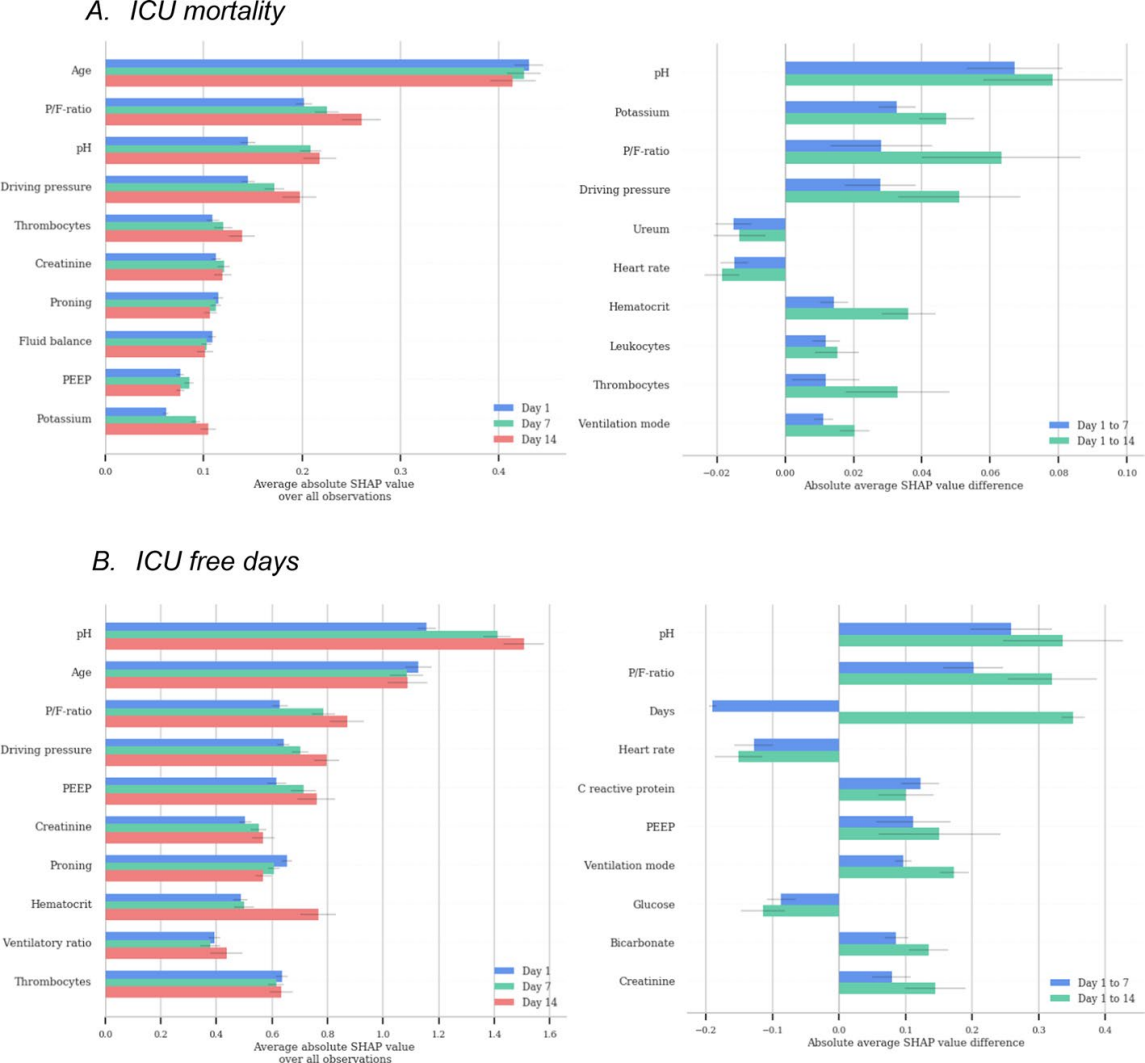
Importance of the top 10 predictors for the prediction of ICU mortality and ICU free days, as well as the difference for predictors over time. **A** ICU mortality. **B** ICU-free days

### Predictor importance

The most important predictors per time point based on the SHAP values are presented in Fig. 1; for ventilatory-free days these plots can be found in Additional file 1: Fig. S4. Furthermore, an unregularized linear model was trained to identify statistically significant relationships between the predictors and each outcome, shown in Additional file 1: Table S3. These predictors largely overlapped with the predictors identified with the SHAP values. Lastly, strongly correlated predictors removed during the data preparation are listed in Additional file 1: Table S4.

**Fig. 2:**
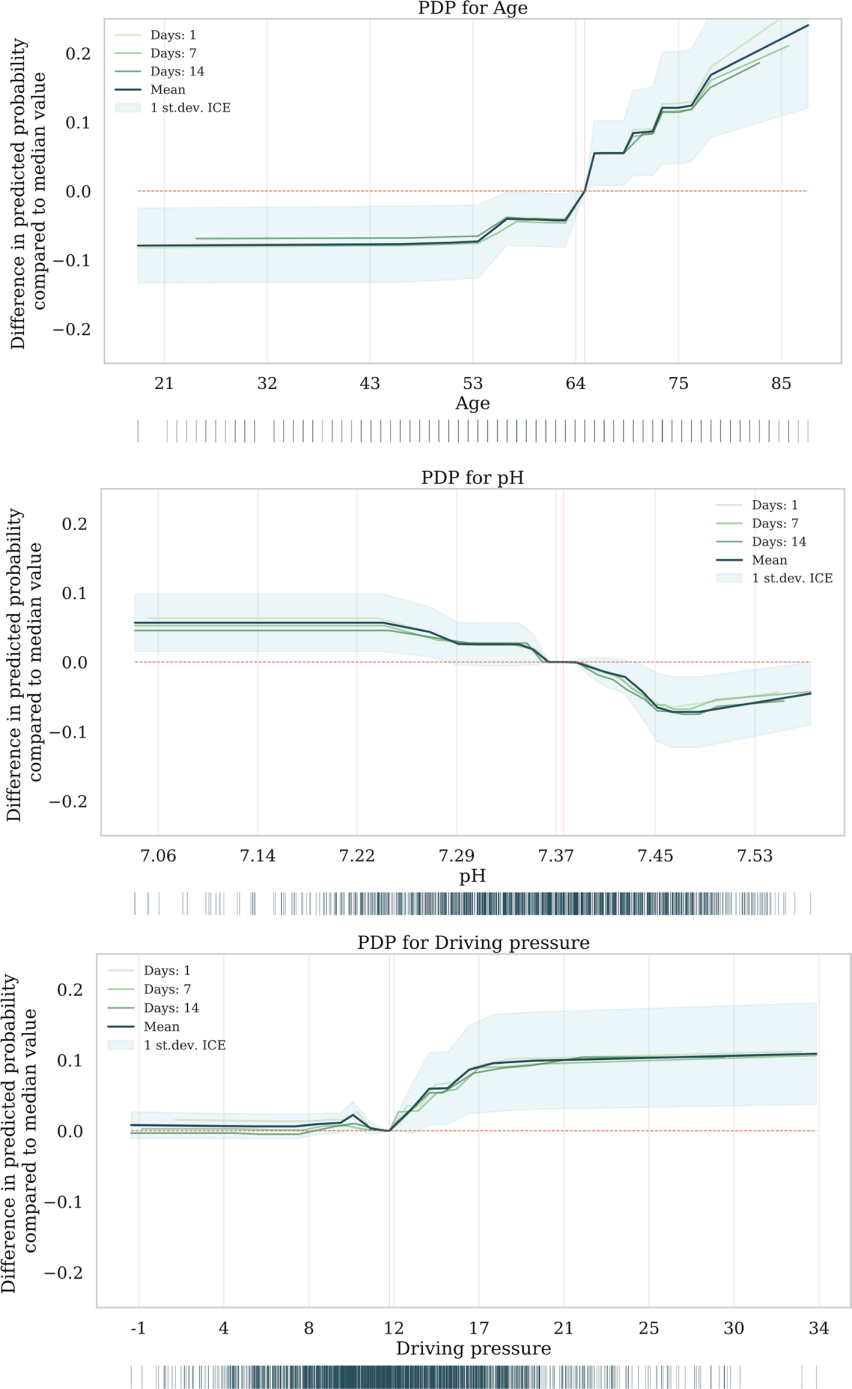
Partial dependence plots. PDP for age, pH, and driving pressure. The median value of all observations is indicated with a red vertical line

Overall, age was the most important demographic predictor of all three outcomes. The PDP shows the relative increase of ICU mortality probability with age, displaying an increase starting at 54 years relative to the median and the steepest increase around the median age of 64 years (Fig. 2). None of the comorbidities showed up as important predictors. Besides age, mechanical ventilation parameters were the most important predictors for all outcomes. Interestingly, fluid balance and medication were neither significantly correlated with outcome, nor among the most important predictors based on their SHAP values. However, medication dosage was unavailable for several hospitals (11 out of 25).

**Fig. 3:**
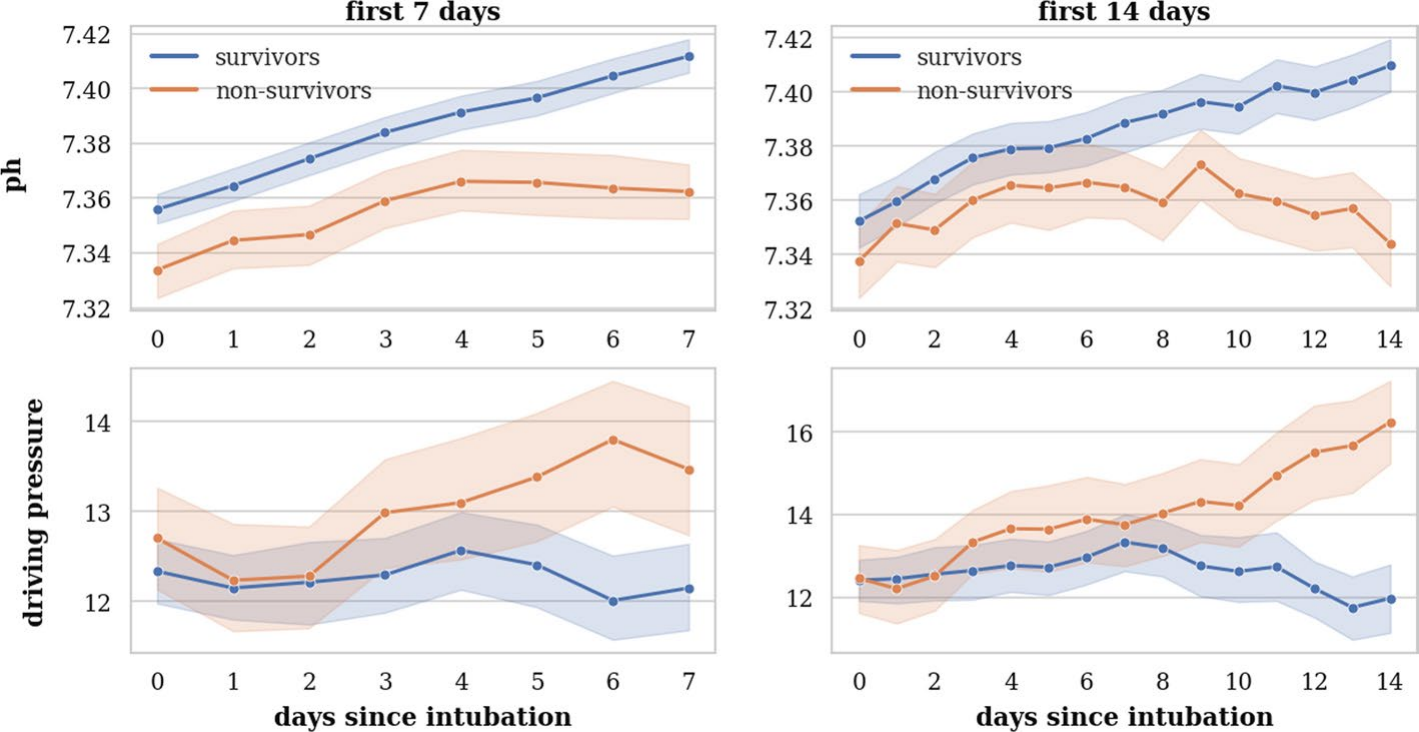
Course of pH and driving pressure. Plots show the course of pH and driving pressure only for patients intubated at least 7 (left) or 14 (right) days, respectively

The pH, P/F ratio and driving pressure were the most important mechanical ventilation predictors for all outcomes; acidaemic conditions, low P/F-ratios, and high driving pressures were associated with a higher probability of mortality. The magnitude and direction of all predictors’ effect can be observed in the SHAP plots in Additional file 1: Figs. S5, S6, and S7. pH was strongly correlated with pCO_2_, while no correlation was found with creatinine, AKI stage, or lactate. The median pH in the PDP falls within the normal range for pH. The course of pH between survivors and non-survivors can be observed in Fig. 3 and shows higher pH in survivors, albeit close to the normal range of pH. Conversely, the average applied driving pressure increased in non-survivors compared to survivors throughout the course of IMV. The PDP shows that probability of ICU mortality increases with the mean driving pressure value in the last 24 h at 1, 7, and 14 days. The PDPs of the other predictors can be found in Additional file 1: Fig. S8.

## Discussion

This study identifies risk factors for ICU mortality, ICU-free days and ventilator-free days throughout the course of invasive mechanical ventilation for critically ill COVID-19 patients. Even though demographics of the COVID-19 population remained similar throughout the first 14 days of IMV, age was consistently the most important demographic risk factor of outcome. pH and respiratory characteristics became increasingly important risk factors throughout the course of IMV.

Risk factors of poor ICU outcome are important to gauge prognosis for a group of patients, in order to scientifically underpin the larger debate of resource allocation in the COVID-19 crisis, and to generate hypotheses to improve our understanding of the disease. Although the exact pathophysiology remains unknown [21], previous work has linked age on admission to poor prognosis in critically ill COVID-19 patients [3, 22]. We now show that increasing age is a consistent risk factor for ICU mortality throughout the course of IMV, with an increase in the relative probability of death starting at 54 years. In addition, age is the most important demographic predictor for ventilator-free days and ICU-free days.

Besides demographics, the presented models are unique in the variety of clinical characteristics incorporated as predictors. From these parameters, pH, *P*/*F* ratio, and driving pressure demonstrate increasing importance over the course of IMV. Studies investigating the role of these predictors in critically ill COVID-19 patients are limited, but did identify pH as an important predictor [23]. No studies are available looking at the role of these predictors throughout the course of ICU admission. In the current study pH is correlated with pCO_2_, while no significant correlation is found with renal function or lactate. Persisting low pH may therefore reflect continued protective ventilation with permissive hypercapnia and serve as a proxy for severity of respiratory illness. Likewise, driving pressure and *P*/*F* ratio may reflect the state of the lung. Whether maintaining a lower pH or high driving pressure may have adverse effects on the body throughout the course of IMV directly remains to be investigated.

### Observational data and future perspectives

While the risk factors identified in this work provide important prognostic insight for clinicians and policy-makers, relationships provide associations rather than causal effects. For causal modeling, however, a thorough understanding of causal pathways is essential. Predictors associated with outcome and intervention under study need to be understood to improve causal inference. In addition, as with any observational dataset, not all relevant predictors may be captured, potentially leading to confounding. This work sheds light on important predictors and fuels the discussion on potential confounders and standardization across EHRs. Lastly, this work generates important insight in relevant predictors that require further study. In line with the lung protective strategy, including acceptance of low pH and high pCO_2_, driving pressure has previously been causally related to outcome [16]. This work elaborates that such a relationship extends beyond the first 24 h of IMV, but remains to be researched further.

### Model performance

Identification of risk factors depends on the goodness of fit of the model, and we show that model performance for ICU mortality [24], as well as for ventilator and ICU days [25], is consistent with the pre-COVID-19 literature. We observe an increase in model performance later throughout the course of IMV, which may indicate that the clinical characteristics better reflect the state of the patient, or the predictors more uniformly relate to the outcome. Ideally, prediction models would be integrated in the EHR and provide clinicians with a personalized mortality prediction at any given time at the bedside. Further investigations are needed to optimize individual predictions to be reliable for clinical decisions with irreversible consequences.

### Strengths and limitations

This paper has several strengths. First of all, this study is unique in both the variety of predictors available per patient and the time-course data included in the models. Moreover, the multicenter data reflect practice differences between centers and improves external validity. Finally, data and code used in this study are available to clinicians and researchers within legal and ethical boundaries [11]. Data sharing is essential to replicate and verify results, compare underlying data and collaborate to foster the understanding of COVID-19.

The present study also comes with limitations. Firstly, removing transferred patients may have introduced bias in the dataset. Transferals may represent a healthier cohort of patients that are fit for transport or a sicker cohort transferred for specialized care such as extracorporeal life support (ECLS). Nonetheless, whenever data from the referring and receiving hospital were available, patient data were connected to limit the number of exclusions. In addition, previous analyses have shown that on admission, transferred patients are similar to non-transferred patients [9]. The DDW represents a relatively unselected sample of patients since all COVID-19 patients from the participating ICUs were included, limiting selection bias. Secondly, observations throughout the time course of IMV may be correlated in the same patient. To prevent leakage of correlated information, however, we keep observations of the same patient in the same split. In addition, predictors may be correlated with each other in the same observation. For this, we removed correlated predictors and we trained decision trees, which are robust to correlations. Moreover, medication dosage was still missing for certain hospitals. When unavailable, values were imputed with the median daily dosage in the training set. Nonetheless, we expect steroids to converge to similar doses in the beginning of admission due to the latest evidence.

## Conclusion

This study trained a set of machine learning algorithms on a large, full-admission cohort of COVID-19 patients to identify the risk factors for ICU mortality, and ventilation- and ICU-free days throughout the course of invasive mechanical ventilation. Consistently, age was the most important demographic risk factor, with an increase in the relative probability of death starting at 54 years. Nonetheless, pH, *P*/*F* ratio, and driving pressure provided increasingly important risk factors over time. These results can be used for prognostication and to provide insight for the debate on resource allocation. In addition, the results of this research serve as a stepping stone for causal inference and individualized predictions research.

## Supplementary Information

**Additional file 1: Figure S1.** Patient selection. **Figure S2.** Selection of observations throughout the course of IMV. **Figure S3.** Nested cross-validation. **Figure S4.** Importance of the top 10 predictors for the prediction of ventilator free days, as well as the difference for predictors over time. **Figure S5.** SHAP plot ICU mortality (XGBoost). **Figure S6.** SHAP plot for ICU free days (XGBoost). **Figure S7.** SHAP plot for ventilator free days (XGBoost). **Figure S8.** PDPs. **Table S1.** Overview of all predictors used in the model with a definition where applicable. **Table S2.** Overall algorithm performance for each of the different outcomes. **Table S3.** Statistical results for a regression model per outcome. **Table S4.** Predictor correlations.

## Data Availability

The dataset supporting the conclusions of this article is available within restrictions imposed by privacy laws and ethics through www.amsterdammedicaldatascience.nl

## Abbreviations

DDW: Dutch data warehouse
IMV: Invasive mechanical ventilation
SHAP: Shapley additive explanations
PDP: Partial dependence plots
