# Supplementary material for: Risk factors for adverse outcomes during mechanical ventilation of 1152 COVID-19 patients: a multicenter machine learning study with highly granular data from the Dutch Data Warehouse
Source: Intensive Care Med Exp. 2021 Jun 28;9:32. doi: 10.1186/s40635-021-00397-5 (PMC8236316; doi:10.1186/s40635-021-00397-5)
Supplement: Supplementary file 1 — Additional file 1: Figure S1. Patient selection. Figure S2. Selection of observations throughout the course of IMV. Figure S3. Nested cross-validation. Figure S4. Importance of the top 10 predictors for the prediction of ventilator free days, as well as the difference for predictors over time. Figure S5. SHAP plot ICU mortality (XGBoost). Figure S6. SHAP plot for ICU free days (XGBoost). Figure S7. SHAP plot for ventilator free days (XGBoost). Figure S8. PDPs. Table S1. Overview of all predictors used in the model with a definition where applicable. Table S2. Overall algorithm performance for each of the different outcomes. Table S3. Statistical results for a regression model per outcome. Table S4. Predictor correlations. [file 40635_2021_397_MOESM1_ESM.docx]

# Additional Files

## **Additional Figure 1.** Patient selection.


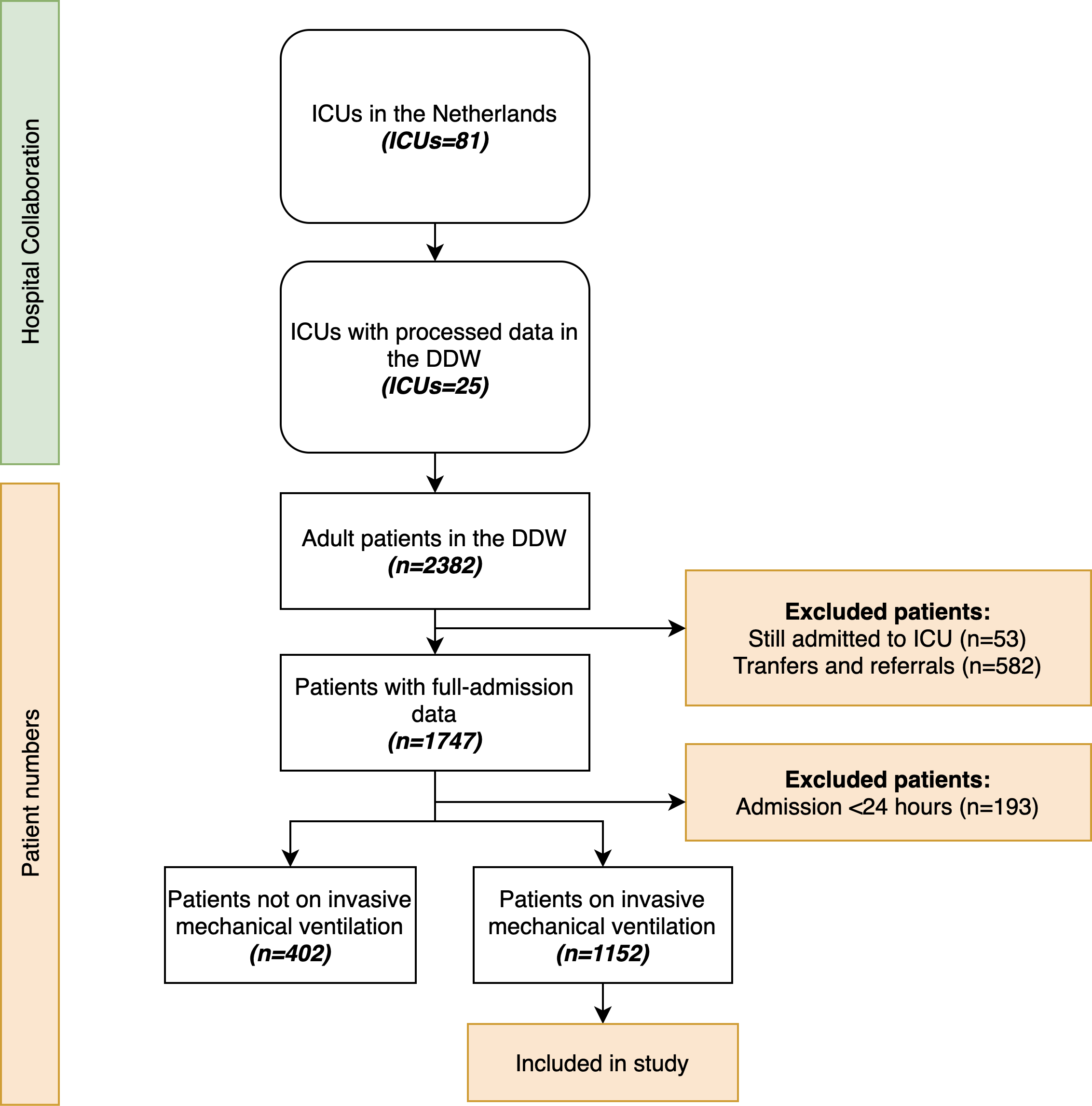


*Inclusion of ICU’s in the Netherlands and patient selection in the study*

*DDW: Dutch Data Warehouse*

## **Additional Figure 2.** Selection of observations throughout the course of IMV


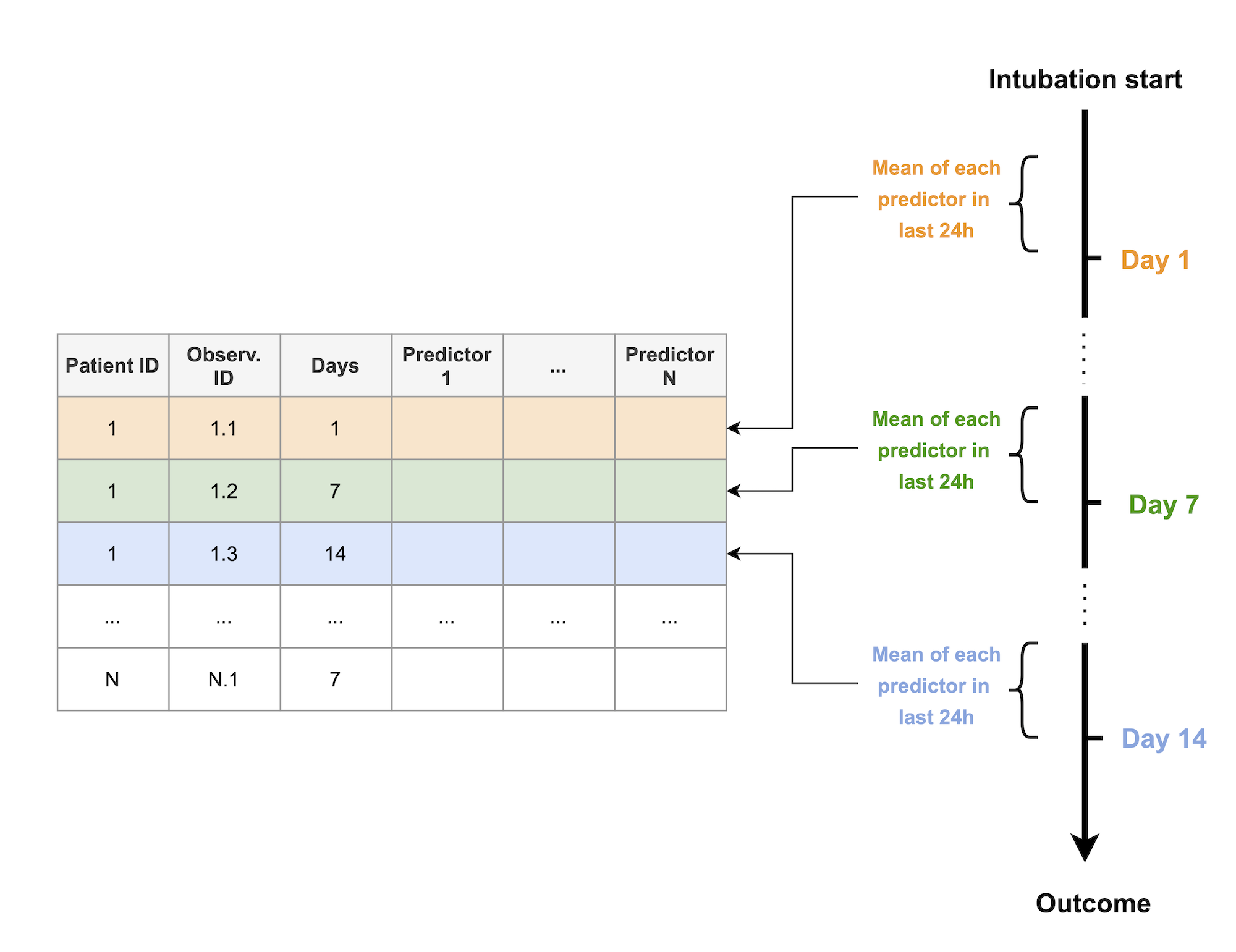


## **Additional Figure 3.** Nested cross-validation

##

##
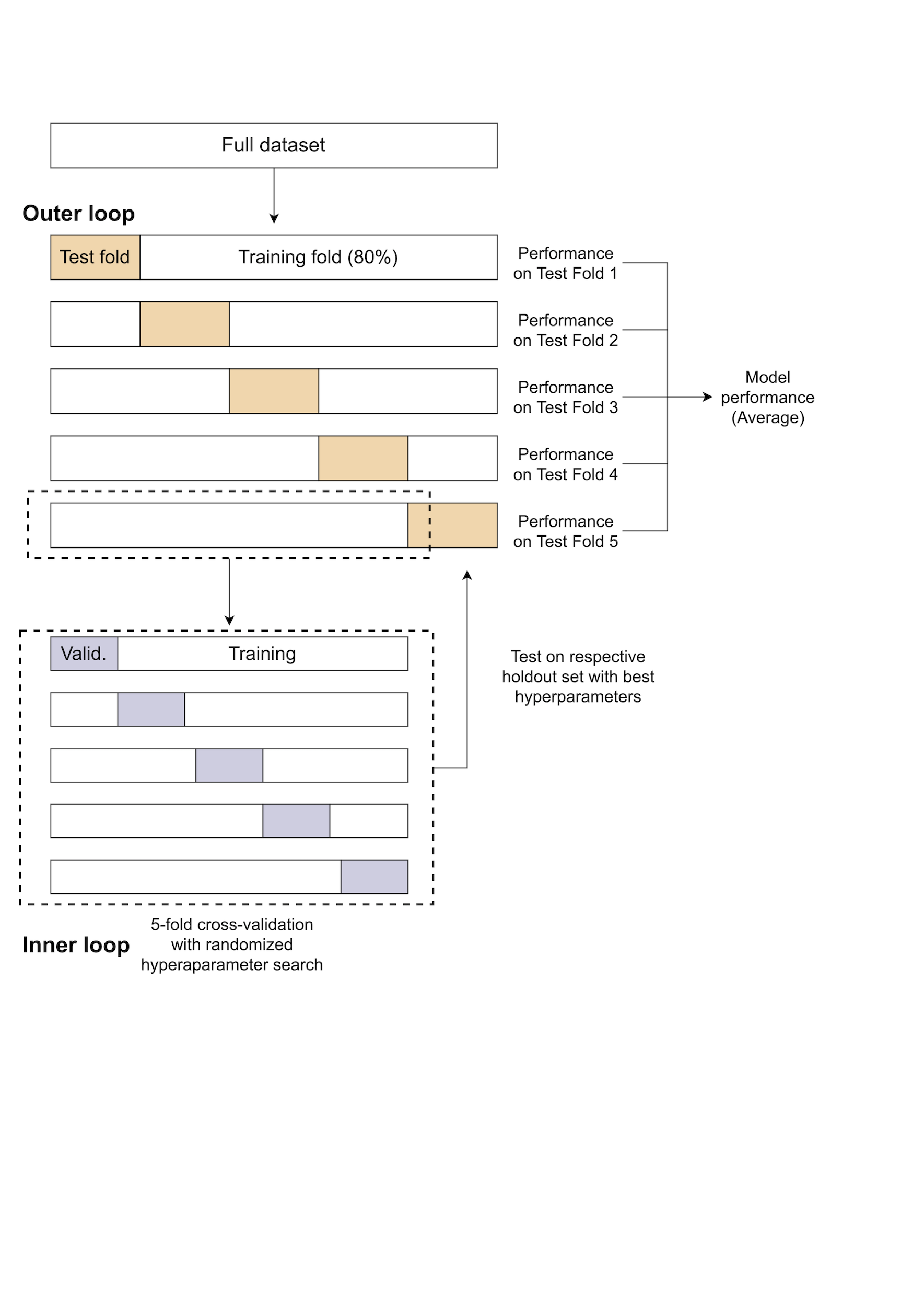


## **Additional Figure 4.** Importance of the top 10 predictors for the prediction of ventilator free days, as well as the difference for predictors over time


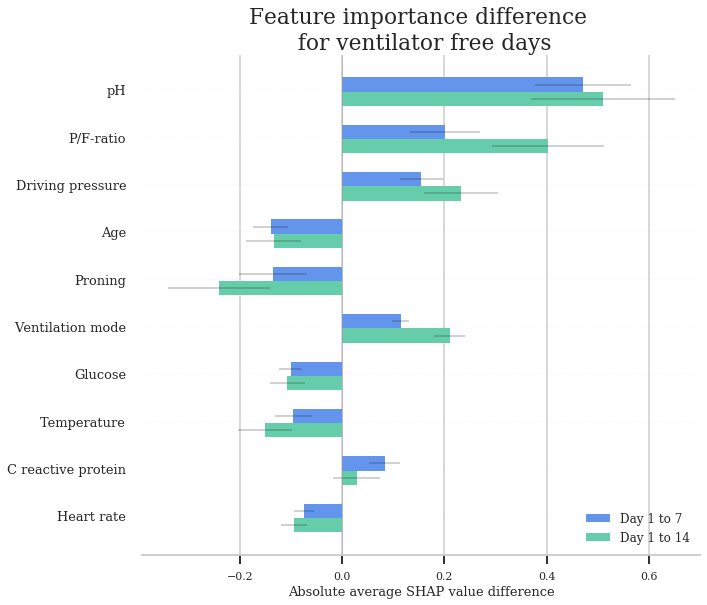

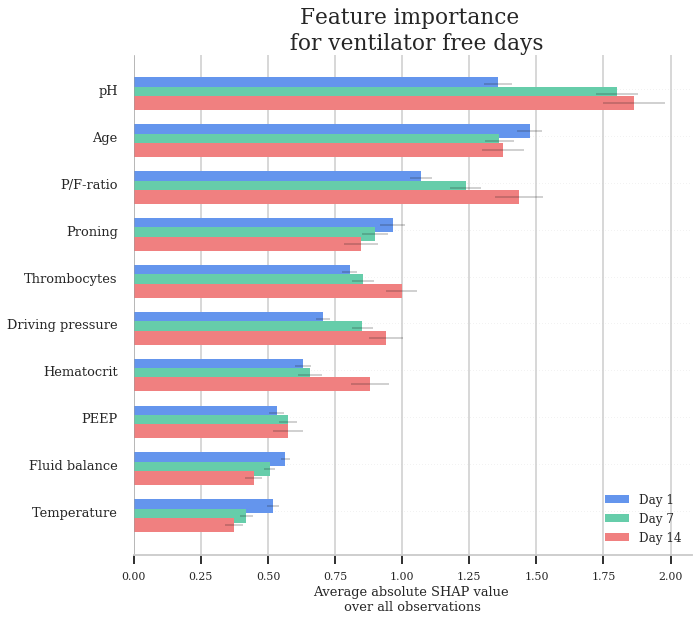


*Overview of predictor importance (left) and relative importance (right) throughout the course of IMV for ventilator free days*

*PEEP: positive end expiratory pressure*

## **Additional Figure 5.** SHAP plot ICU mortality (XGBoost)

##
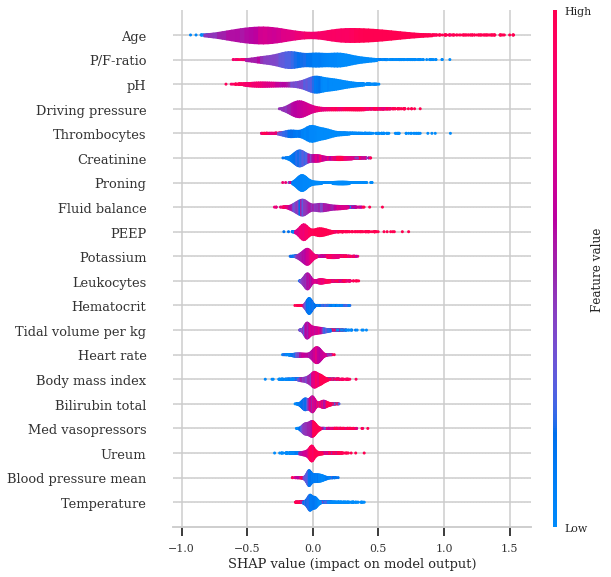


##

*SHAP plot indicating the impact of the predictors on the model output for all observations (at 1, 7, and 14 days after IMV). Top 20 predictors are shown.*

## **Additional Figure 6.** SHAP plot for ICU free days (XGBoost)

##

##
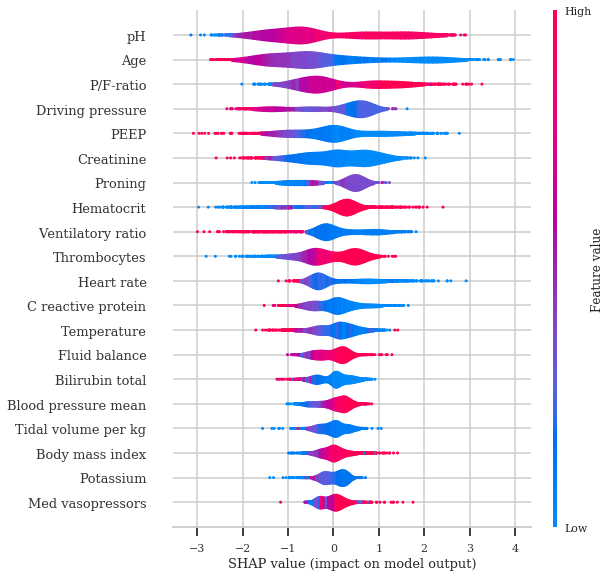


*SHAP plot indicating the impact of the predictors on the model output for all observations (at 1, 7, and 14 days after IMV). Top 20 predictors are shown.*

##

## **Additional Figure 7.** SHAP plot for ventilator free days (XGBoost)

##

##
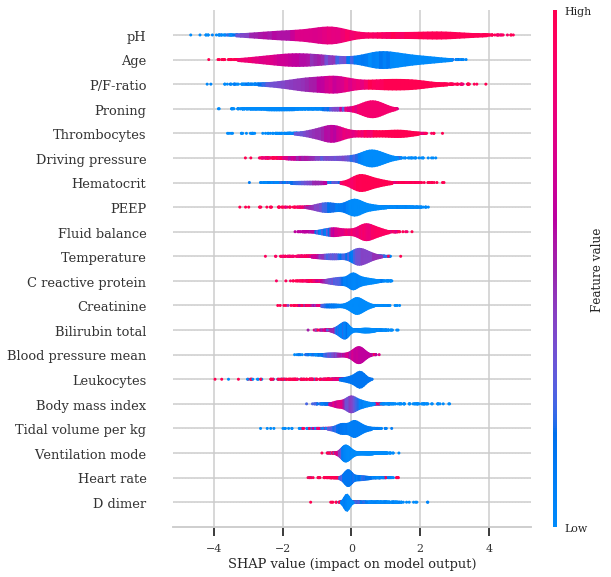


*SHAP plot indicating the impact of the predictors on the model output for all observations (at 1, 7, and 14 days after IMV). Top 20 predictors are shown.*

## **Additional Figure 8.** PDPs


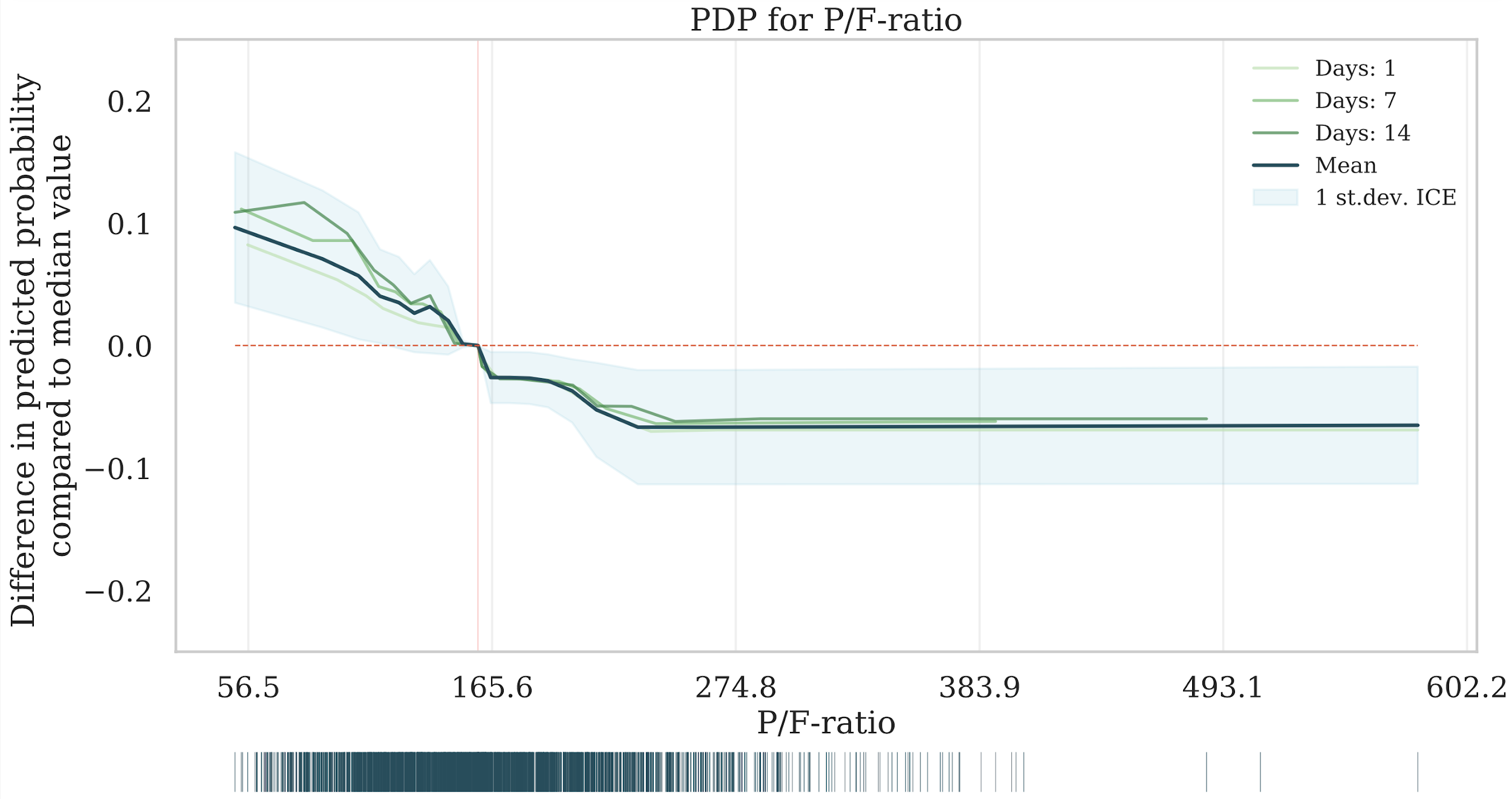


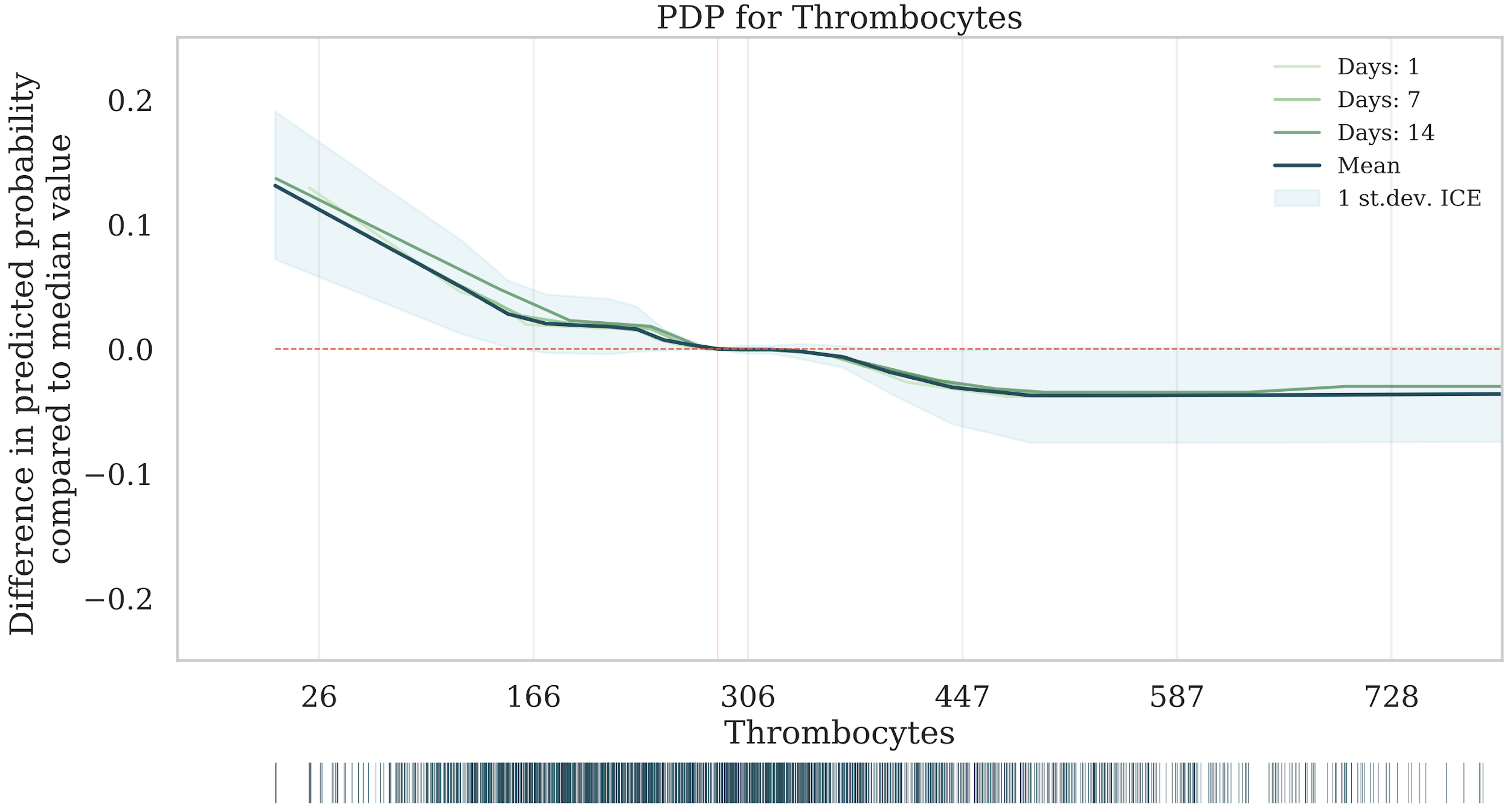


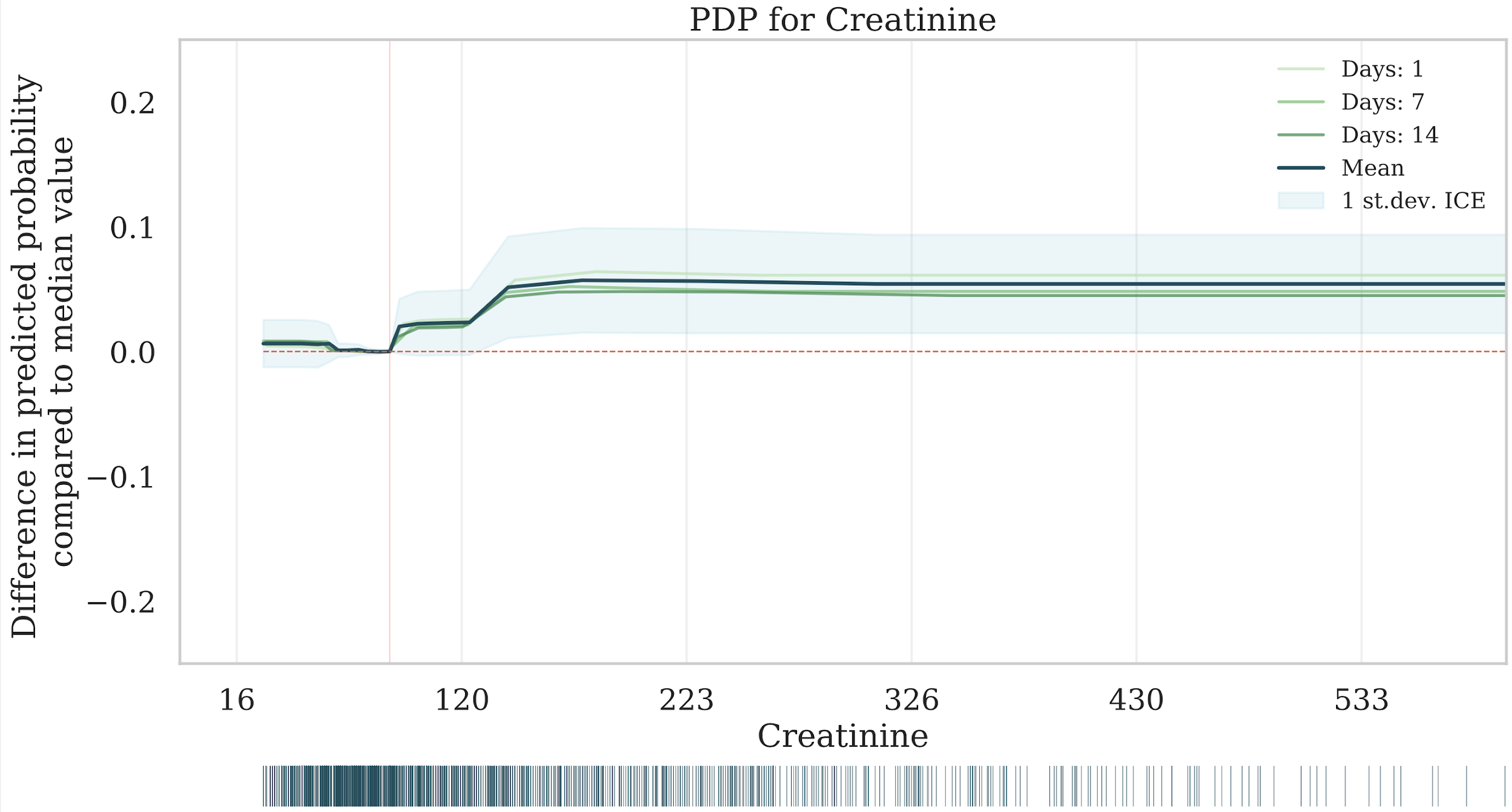


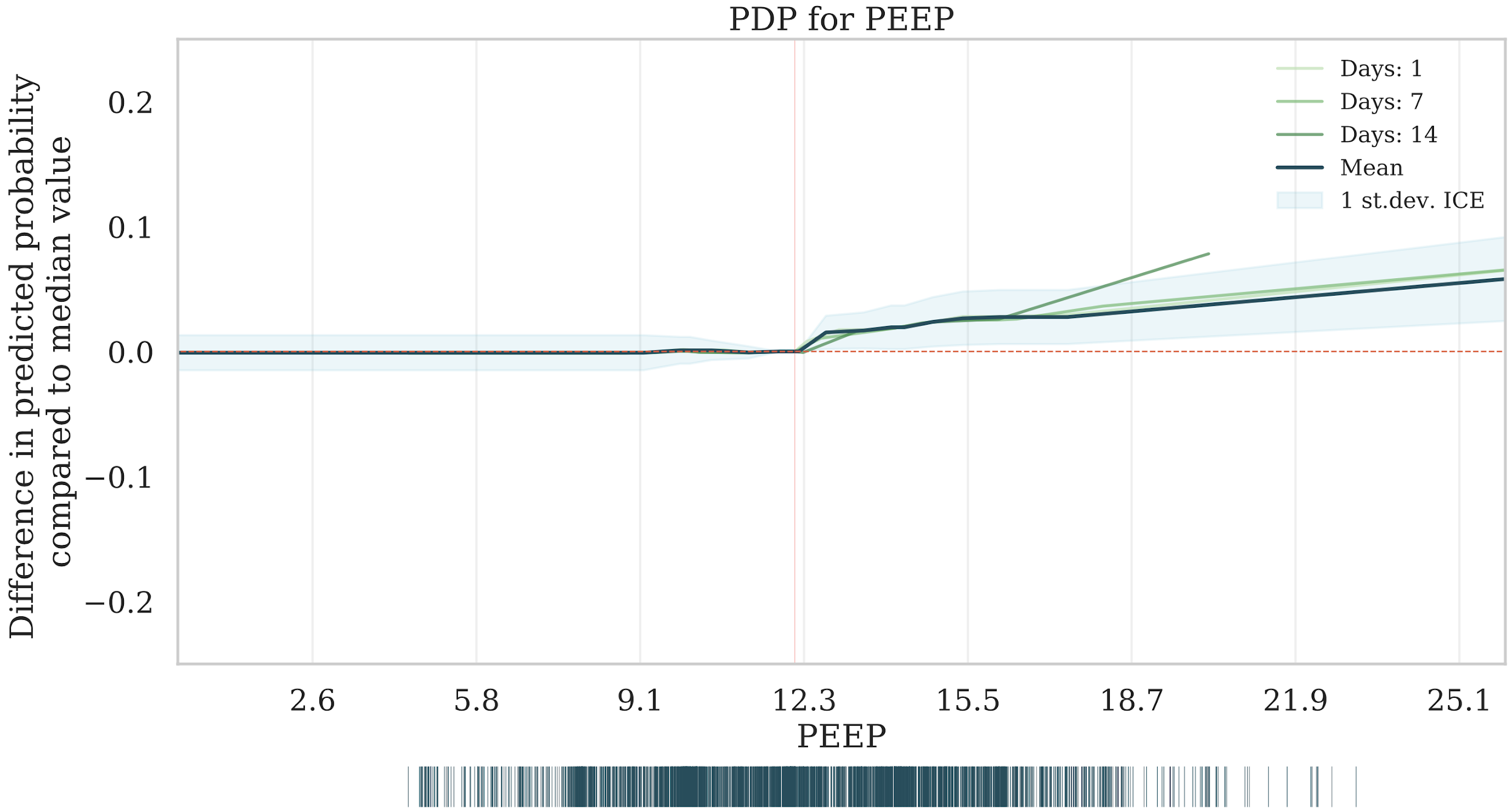


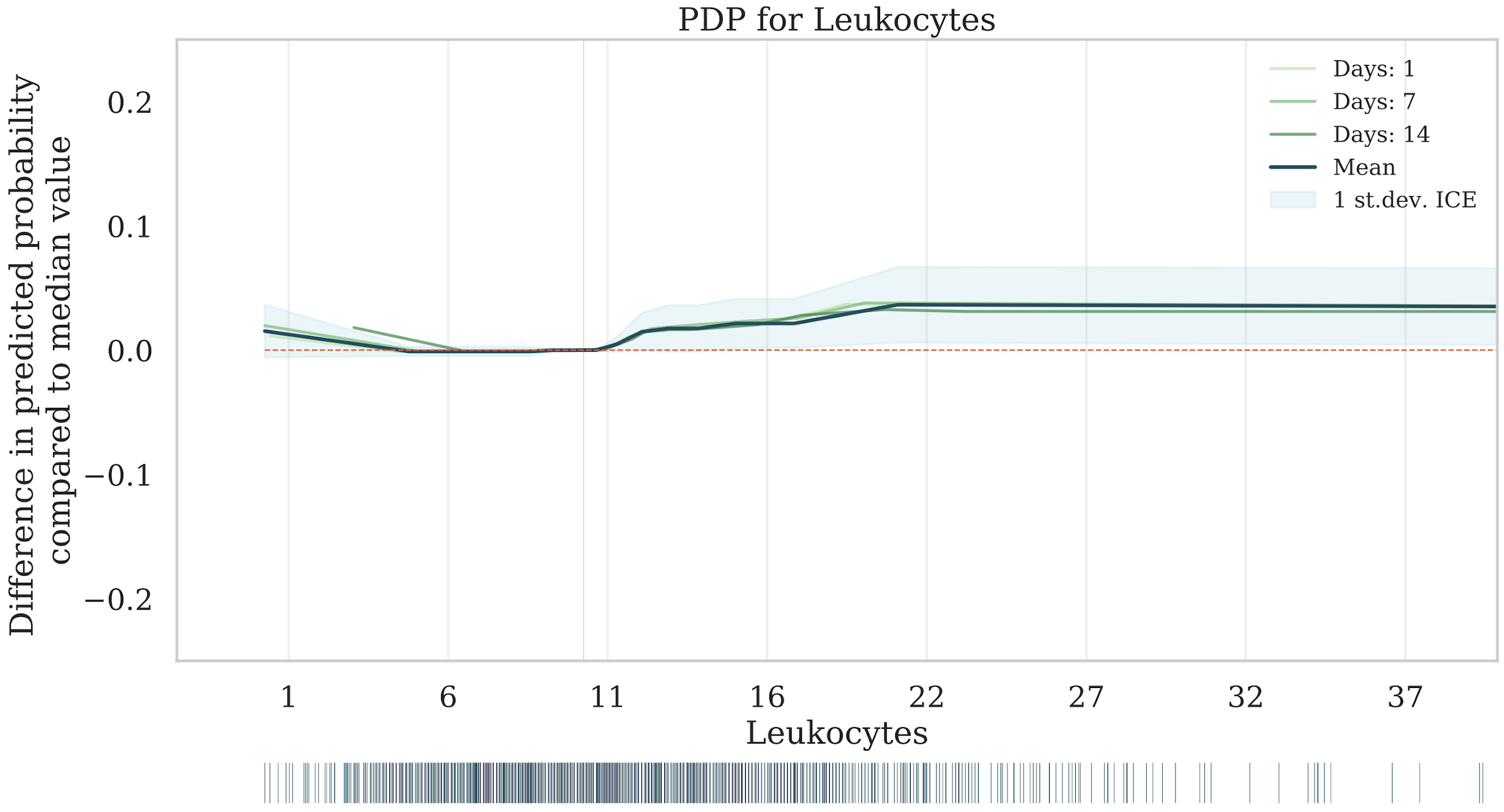


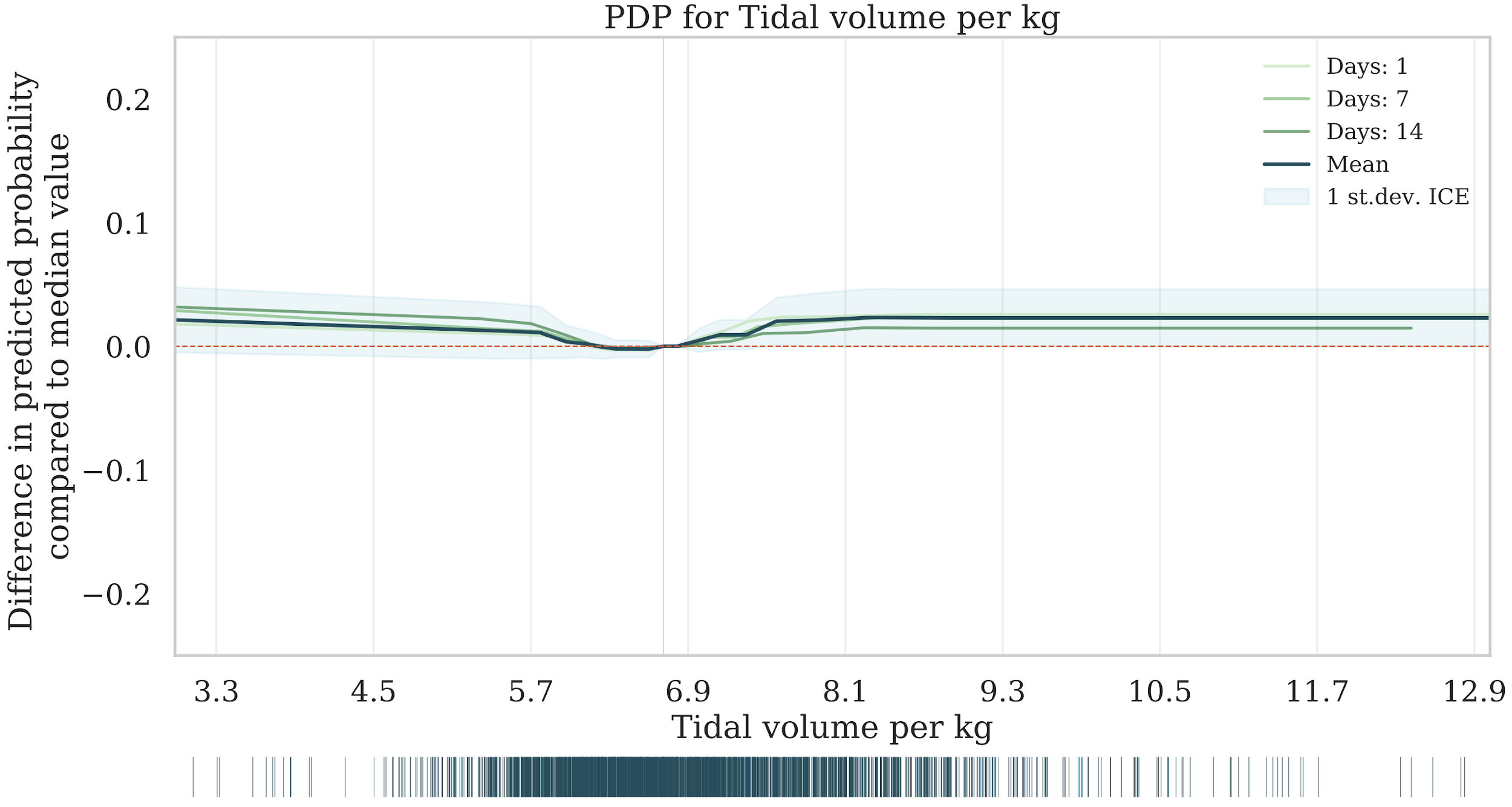


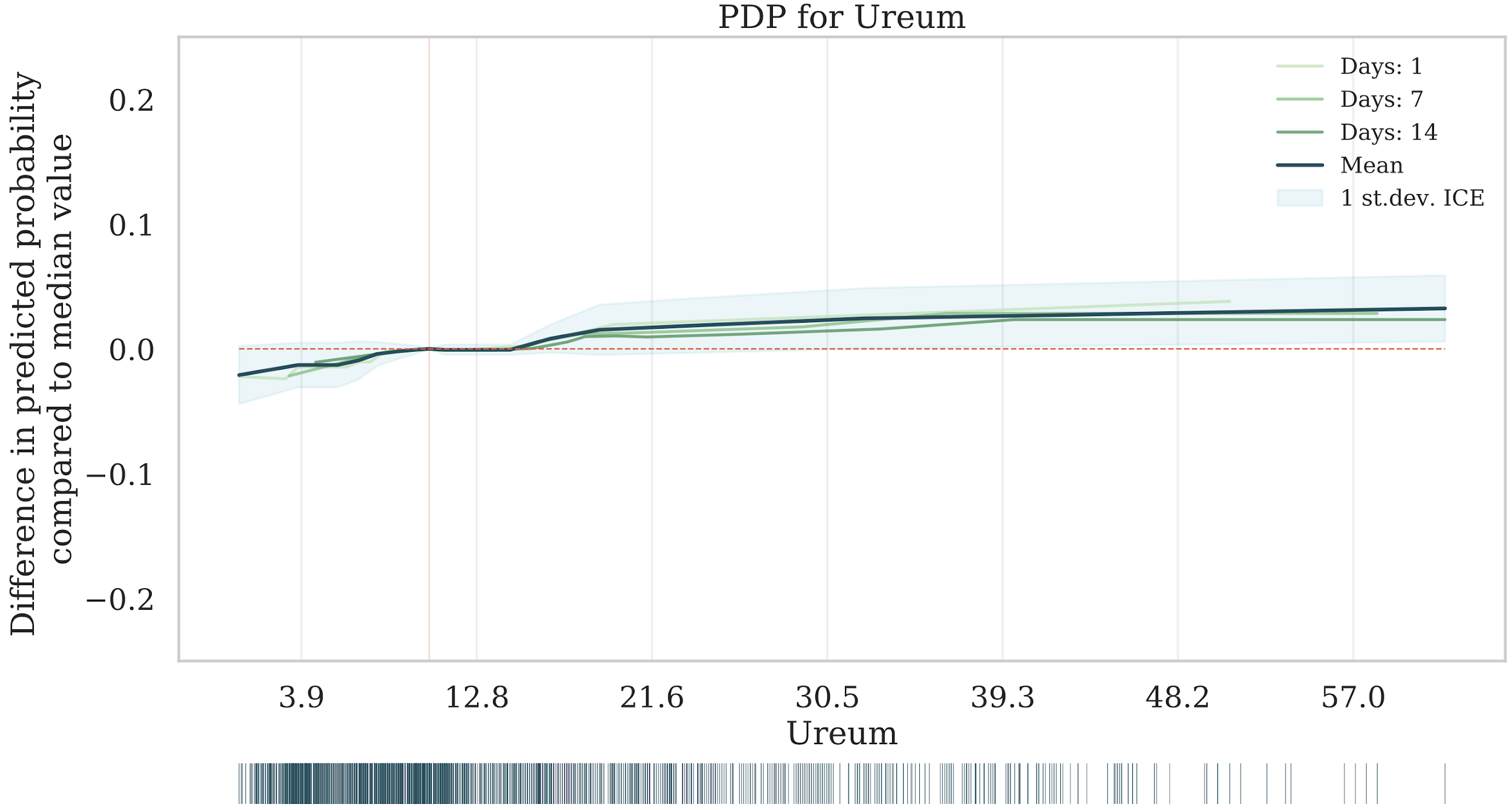


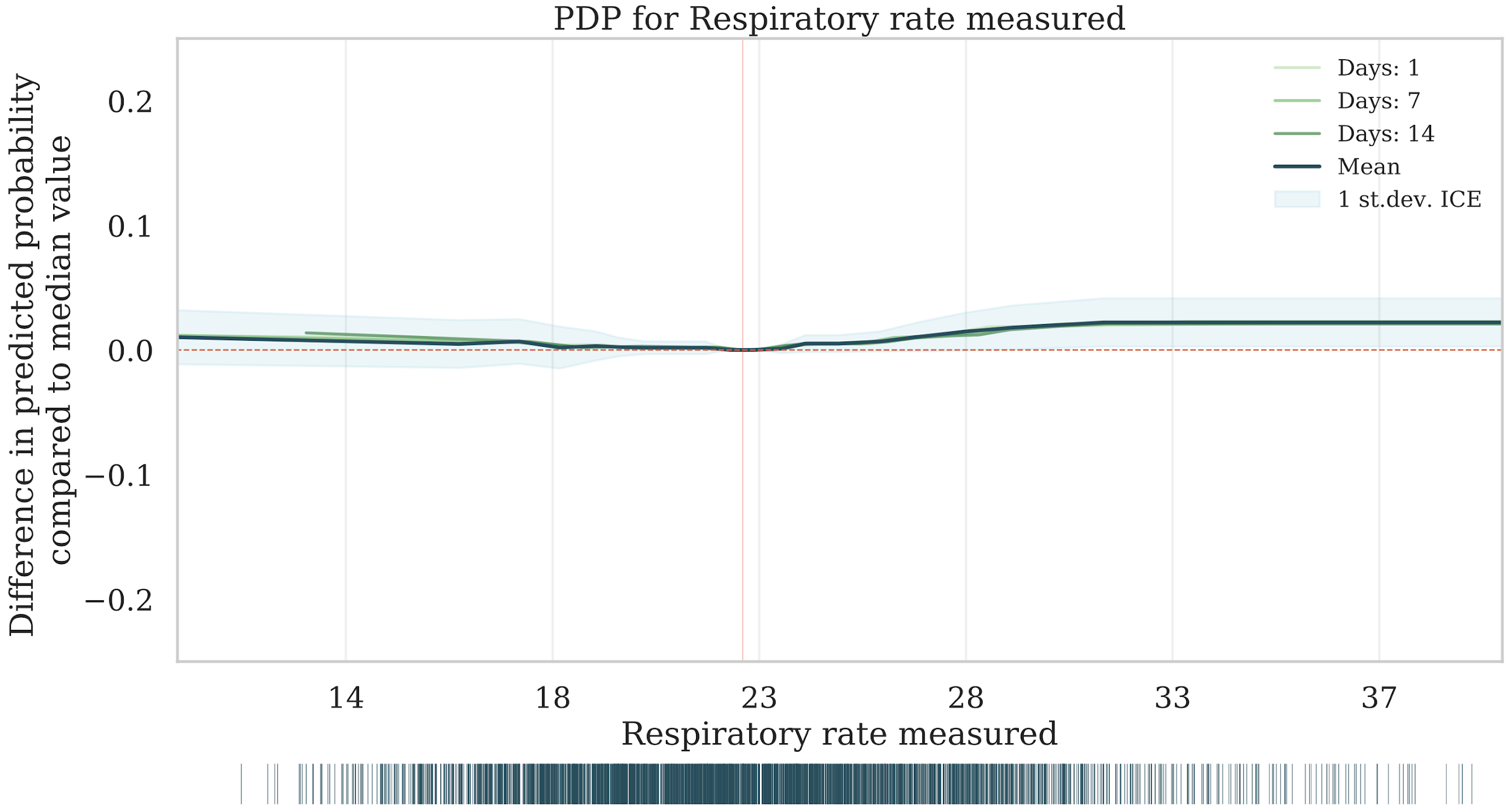


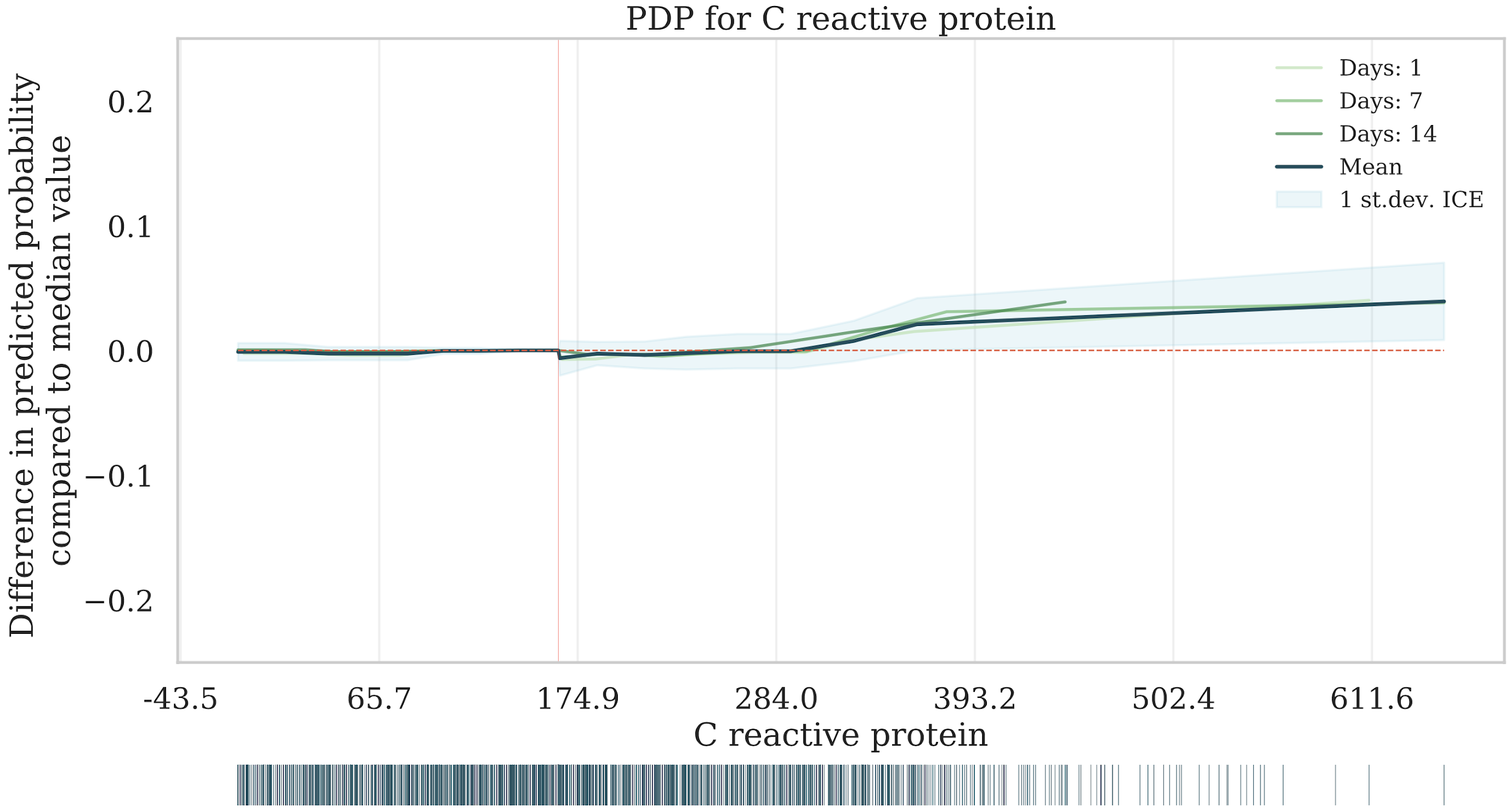


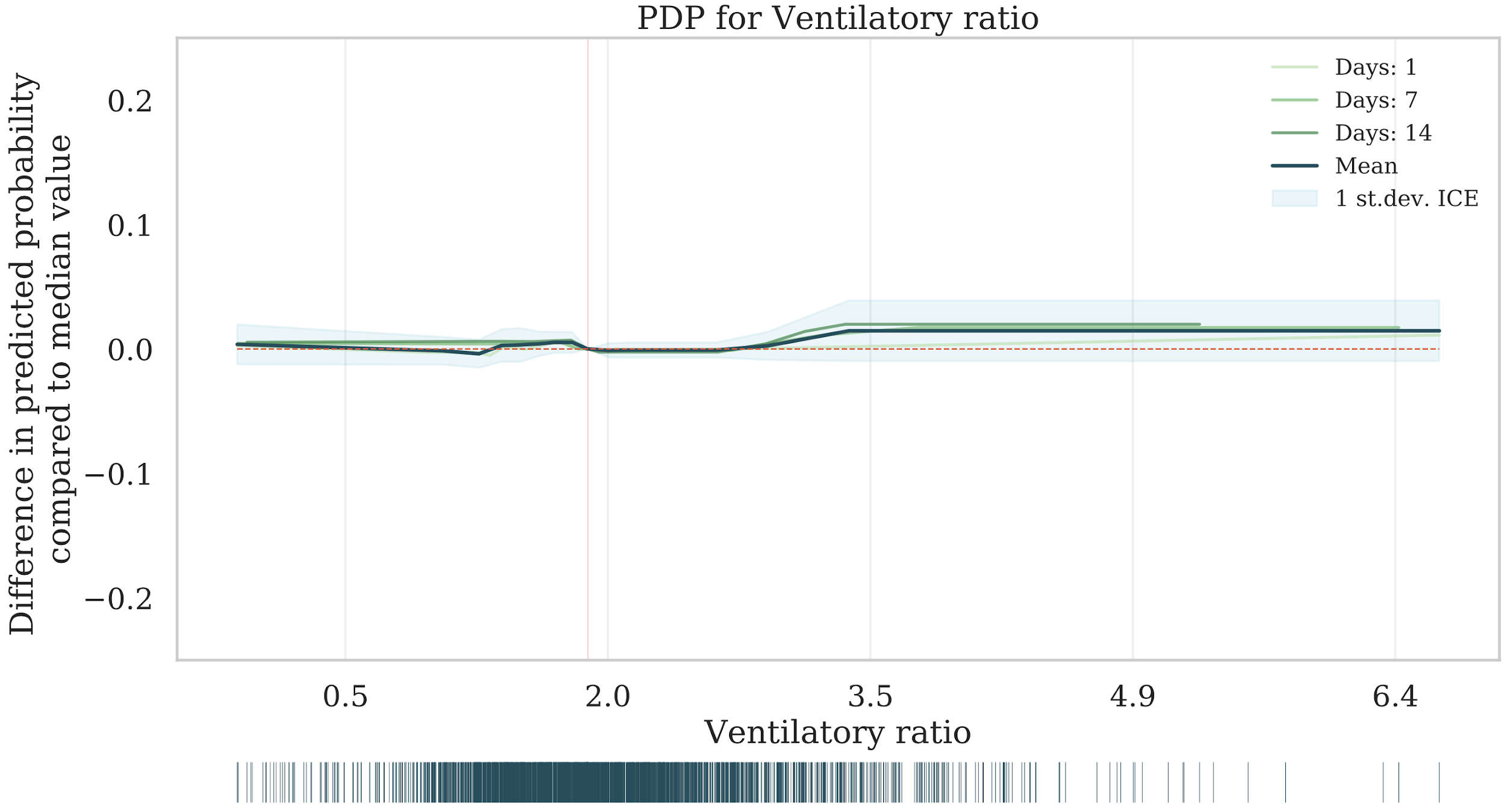


##

## **Additional Table 1.** Overview of all predictors used in the model with a definition where applicable

| **Predictors** | **Amato et al.** | **Severity scores^a^** | **Intensivist panel** | **Definition where applicable** |
| --- | --- | --- | --- | --- |
| Acute kidney injury |  |  | Yes | KDIGO staging |
| Age | Yes | Yes |  |  |
| Albumin |  | Yes |  |  |
| Bicarbonate |  | Yes |  |  |
| Bilirubin |  | Yes |  |  |
| Mean blood pressure |  |  |  |  |
| Body mass index (BMI) |  |  | Yes | Weight/height^2^ |
| C-reactive protein |  |  | Yes |  |
| Creatinine |  | Yes |  |  |
| D-dimer |  |  | Yes |  |
| Days |  |  | Yes | Number of days since the beginning of intubation |
| Driving pressure | Yes |  |  | The recorded static compliance or the tidal volume/(plateau pressure - peep) |
| FiO_2_ | Yes | Yes |  | Fraction of inspired oxygen |
| Fluid balance sum |  | Yes |  | Fluid in = positive balance  Fluid out = negative balance |
| Glucose |  | Yes |  |  |
| Acute Kidney Injury |  |  | Yes | Defined according to the KDIGO staging |
| Comorbidity - Cancer |  |  | Yes | Neoplasm or hematologic malignancy |
| Comorbidity - Diabetes |  |  | Yes |  |
| Comorbidity - Immune insufficiency |  |  | Yes |  |
| Comorbidity - Respiratory insufficiency/COPD |  |  | Yes | COPD or respiratory insufficiency |
| Comorbidity - Renal failure |  |  | Yes | Chronic dialysis or chronic renal insufficiency |
| Heart rate |  | Yes |  |  |
| Hematocrit |  | Yes |  |  |
| Gender |  |  | Yes |  |
| Leukocytes |  | Yes |  |  |
| Lung compliance static | Yes |  |  |  |
| Mean arterial blood pressure |  | Yes |  |  |
| Medication - Steroids   - Hydrocortisone - Prednisolone - Betamethasone - Dexamethasone - Betamethasone - Methylprednisolone - Prednisolone - Prednisone - Hydrocortisone - Cortisone |  |  |  | Mean total dose administered |
| Medication - Cardiovascular   - Norepinephrine - Dopamine - Dobutamine - Epinephrine |  | Yes |  | Mean total dose administered |
| P/F-ratio | Yes |  |  |  |
| PCO_2_ (art.) | Yes | Yes |  | Arterial partial pressure of CO_2_ |
| PEEP | Yes |  |  | Applied PEEP |
| Plateau pressure | Yes |  |  | In volume control mode |
| pH | Yes | Yes |  |  |
| PO_2_ (Art.) | Yes | Yes |  | Arterial partial pressure of O_2_ |
| Position |  |  | Yes | Number of hours since the patient was last in prone position |
| Potassium |  | Yes |  |  |
| Respiratory rate | Yes | Yes |  |  |
| Sodium |  | Yes |  |  |
| Systolic blood pressure |  | Yes |  |  |
| Temperature |  | Yes |  |  |
| Thrombocytes |  | Yes |  |  |
| Tidal volume | Yes |  |  |  |
| Ureum |  | Yes |  |  |
| Ventilatory ratio |  |  | Yes | Minute volume * PCO_2_ / (predicted body weight * 100 * 37.5) |
| Ventilation mode |  |  | Yes | Percentage of the last 24h spent in controlled vent mode |

## **Additional Table 2.** Overall algorithm performance for each of the different outcomes

*ICU Mortality*

|  | AUROC | AP | Calibration Loss | Brier Score |
| --- | --- | --- | --- | --- |
| Decision Tree | 0.695 ± 0.027 | 0.489 ± 0.018 | 0.009 ± 0.004 | 0.189 ± 0.012 |
| LR | 0.744 ± 0.023 | 0.56 ± 0.042 | **0.008 ± 0.002** | 0.176 ± 0.008 |
| XGBoost | **0.774 ± 0.023** | **0.621 ± 0.056** | 0.010 ± 0.004 | **0.165 ± 0.007** |

*ICU Free Days*

|  | R2 | Median Abs. Error | Mean Abs. Error | Mean Sq. Error |
| --- | --- | --- | --- | --- |
| Lasso | 0.118 ± 0.009 | 9.08 ± 0.10 | 9.01 ± 0.17 | 99.0 ± 3.3 |
| Ridge | 0.179 ± 0.050 | 7.75 ± 0.32 | 8.16 ± 0.26 | 92.0 ± 3.8 |
| XGBoost | **0.212 ± 0.028** | **7.51 ± 0.18** | **8.01 ± 0.10** | **88.5 ± 2.5** |

*Ventilator Free Days*

|  | R2 | Median Abs. Error | Mean Abs. Error | Mean Sq. Error |
| --- | --- | --- | --- | --- |
| Lasso | 0.169 ± 0.015 | 9.39 ± 0.16 | 8.84 ± 0.09 | 98.3 ± 1.3 |
| Ridge | 0.217 ± 0.038 | 7.76 ± 0.13 | 8.20 ± 0.09 | 92.6 ± 3.3 |
| XGBoost | **0.250 ± 0.033** | **7.72 ± 0.09** | **8.05 ± 0.08** | **88.7± 2.6** |

*Overall model performance for each of the different outcomes for all observations (at 1, 7, and 14 days after IMV).*

*AUROC: area under the receiver operating characteristic, AP: area under the precision recall curve, median abs. error = median absolute error, mean sq. error: mean squared error*

## **Additional Table 3.** Statistical results for a regression model per outcome

*ICU Mortality (Logistic Regression)*

|  | coef | std err | z | P>\|z\| | [0.025 | 0.975] |
| --- | --- | --- | --- | --- | --- | --- |
| Const | 31.8722 | 7.865 | 4.052 | 0.000 | 16.457 | 47.287 |
| Age | 0.0528 | 0.006 | 8.589 | 0.000 | 0.041 | 0.065 |
| Thrombocytes | -0.0025 | 0.000 | -5.388 | 0.000 | -0.003 | -0.002 |
| Ph | -4.3494 | 1.079 | -4.033 | 0.000 | -6.463 | -2.235 |
| P/f-ratio | -0.0054 | 0.001 | -4.528 | 0.000 | -0.008 | -0.003 |
| Driving pressure | 0.0562 | 0.015 | 3.790 | 0.000 | 0.027 | 0.085 |
| Is male | 0.4341 | 0.135 | 3.227 | 0.001 | 0.170 | 0.698 |
| Has aki comorb yes | -0.6457 | 0.197 | -3.270 | 0.001 | -1.033 | -0.259 |
| Proning | -0.0028 | 0.001 | -3.122 | 0.002 | -0.004 | -0.001 |
| Hematocrit | -3.9066 | 1.264 | -3.090 | 0.002 | -6.385 | -1.428 |
| Temperature | -0.2024 | 0.067 | -3.012 | 0.003 | -0.334 | -0.071 |
| Heart rate | 0.0103 | 0.004 | 2.703 | 0.007 | 0.003 | 0.018 |
| Ventilatory ratio | -0.3355 | 0.127 | -2.643 | 0.008 | -0.584 | -0.087 |
| Has renal comorb yes | 0.7540 | 0.293 | 2.578 | 0.010 | 0.181 | 1.327 |
| Leukocytes | 0.0270 | 0.011 | 2.447 | 0.014 | 0.005 | 0.049 |
| Sodium | 0.0091 | 0.004 | 2.071 | 0.038 | 0.000 | 0.018 |
| Peep | 0.0385 | 0.020 | 1.962 | 0.050 | 0.000 | 0.077 |
| C reactive protein | 0.0011 | 0.001 | 1.929 | 0.054 | 0.000 | 0.002 |
| Days | 0.0349 | 0.018 | 1.907 | 0.057 | -0.001 | 0.071 |
| Tidal volume per kg | 0.1091 | 0.064 | 1.718 | 0.086 | -0.015 | 0.234 |
| Body mass index | 0.0184 | 0.012 | 1.558 | 0.119 | -0.005 | 0.042 |
| Has imm insuf comorb yes | -0.3559 | 0.237 | -1.505 | 0.132 | -0.820 | 0.108 |
| Ureum | 0.0132 | 0.009 | 1.424 | 0.154 | -0.005 | 0.031 |
| Glucose | 0.0365 | 0.026 | 1.389 | 0.165 | -0.015 | 0.088 |
| Respiratory rate measured | 0.0215 | 0.016 | 1.319 | 0.187 | -0.010 | 0.053 |
| Med steroids | -0.0014 | 0.001 | -1.313 | 0.189 | -0.003 | 0.001 |
| Potassium | 0.1293 | 0.110 | 1.171 | 0.242 | -0.087 | 0.346 |
| Has cancer comorb yes | 0.4259 | 0.392 | 1.086 | 0.278 | -0.343 | 1.195 |
| Bicarbonate | 0.0124 | 0.014 | 0.866 | 0.387 | -0.016 | 0.040 |
| Blood pressure mean | -0.0055 | 0.007 | -0.786 | 0.432 | -0.019 | 0.008 |
| Med vasopressors | 0.0011 | 0.001 | 0.784 | 0.433 | -0.002 | 0.004 |
| Has lung comorb yes | 0.1334 | 0.184 | 0.726 | 0.468 | -0.227 | 0.493 |
| Fluid balance | 0.0000 | 0.000 | -0.660 | 0.509 | 0.000 | 0.000 |
| Albumin | 0.0087 | 0.015 | 0.593 | 0.553 | -0.020 | 0.037 |
| Ventilation mode | -0.0724 | 0.154 | -0.471 | 0.638 | -0.374 | 0.229 |
| Bilirubin total | -0.0007 | 0.005 | -0.129 | 0.897 | -0.011 | 0.009 |
| Has diabetes comorb yes | -0.0124 | 0.145 | -0.086 | 0.932 | -0.297 | 0.272 |
| D dimer | 0.0000 | 0.000 | 0.029 | 0.977 | 0.000 | 0.000 |
| Creatinine | 0.0000 | 0.001 | 0.021 | 0.984 | -0.001 | 0.001 |

*ICU free days (OLS)*

|  | coef | std err | t | P>\|t\| | [0.025 | 0.975] |
| --- | --- | --- | --- | --- | --- | --- |
| Const | -131.9269 | 30.890 | -4.271 | 0.000 | -192.502 | -71.351 |
| Has lung comorb yes | -2.6266 | 0.731 | -3.592 | 0.000 | -4.061 | -1.193 |
| Is male | -2.0705 | 0.505 | -4.097 | 0.000 | -3.062 | -1.079 |
| PEEP | -0.3768 | 0.076 | -4.986 | 0.000 | -0.525 | -0.229 |
| Driving pressure | -0.2365 | 0.058 | -4.075 | 0.000 | -0.350 | -0.123 |
| Age | -0.1544 | 0.021 | -7.375 | 0.000 | -0.195 | -0.113 |
| Thrombocytes | 0.0083 | 0.002 | 4.890 | 0.000 | 0.005 | 0.012 |
| Hematocrit | 21.1959 | 4.871 | 4.351 | 0.000 | 11.643 | 30.749 |
| pH | 24.3680 | 4.256 | 5.725 | 0.000 | 16.021 | 32.715 |
| Days | -0.2411 | 0.072 | -3.350 | 0.001 | -0.382 | -0.100 |
| Heart rate | -0.0449 | 0.015 | -3.021 | 0.003 | -0.074 | -0.016 |
| Proning | 0.0097 | 0.003 | 2.931 | 0.003 | 0.003 | 0.016 |
| P/F-ratio | 0.0122 | 0.004 | 2.939 | 0.003 | 0.004 | 0.020 |
| C reactive protein | -0.0062 | 0.002 | -2.688 | 0.007 | -0.011 | -0.002 |
| Has renal comorb yes | -3.1989 | 1.241 | -2.578 | 0.010 | -5.633 | -0.765 |
| Glucose | -0.2380 | 0.105 | -2.266 | 0.024 | -0.444 | -0.032 |
| Med steroids | 0.0070 | 0.003 | 2.248 | 0.025 | 0.001 | 0.013 |
| Temperature | -0.5645 | 0.259 | -2.177 | 0.030 | -1.073 | -0.056 |
| Has diabetes comorb yes | 1.2532 | 0.580 | 2.160 | 0.031 | 0.116 | 2.391 |
| Creatinine | -0.0057 | 0.003 | -2.043 | 0.041 | -0.011 | 0.000 |
| Sodium | -0.0244 | 0.013 | -1.859 | 0.063 | -0.050 | 0.001 |
| Potassium | 0.8032 | 0.436 | 1.843 | 0.065 | -0.051 | 1.658 |
| Has cancer comorb yes | -2.4704 | 1.590 | -1.554 | 0.120 | -5.588 | 0.647 |
| Leukocytes | -0.0646 | 0.045 | -1.445 | 0.149 | -0.152 | 0.023 |
| Bicarbonate | -0.0749 | 0.057 | -1.321 | 0.187 | -0.186 | 0.036 |
| Blood pressure mean | 0.0314 | 0.026 | 1.200 | 0.230 | -0.020 | 0.083 |
| Tidal volume per kg | -0.2665 | 0.236 | -1.131 | 0.258 | -0.729 | 0.196 |
| Med vasopressors | 0.0050 | 0.006 | 0.886 | 0.376 | -0.006 | 0.016 |
| Body mass index | 0.0374 | 0.046 | 0.821 | 0.412 | -0.052 | 0.127 |
| Fluid balance | 0.0000 | 0.000 | 0.545 | 0.586 | 0.000 | 0.000 |
| Bilirubin total | -0.0099 | 0.021 | -0.469 | 0.639 | -0.051 | 0.032 |
| Ventilation mode | -0.2659 | 0.602 | -0.441 | 0.659 | -1.447 | 0.915 |
| D dimer | -0.0001 | 0.000 | -0.347 | 0.728 | 0.000 | 0.000 |
| Has aki comorb yes | 0.2600 | 0.783 | 0.332 | 0.740 | -1.275 | 1.795 |
| Albumin | 0.0088 | 0.056 | 0.158 | 0.874 | -0.101 | 0.118 |
| Has imm insuf comorb yes | 0.1128 | 0.897 | 0.126 | 0.900 | -1.647 | 1.873 |
| Ventilatory ratio | 0.0617 | 0.507 | 0.122 | 0.903 | -0.932 | 1.055 |
| Respiratory rate measured | 0.0051 | 0.063 | 0.082 | 0.935 | -0.118 | 0.128 |
| Ureum | 0.0022 | 0.037 | 0.058 | 0.954 | -0.070 | 0.075 |

*Vent Free Days (OLS)*

|  | coef | std err | t | P>\|t\| | [0.025 | 0.975] |
| --- | --- | --- | --- | --- | --- | --- |
| Const | -185.9015 | 31.183 | -5.962 | 0.000 | -247.052 | -124.751 |
| Age | -0.1569 | 0.021 | -7.424 | 0.000 | -0.198 | -0.115 |
| Thrombocytes | 0.0095 | 0.002 | 5.559 | 0.000 | 0.006 | 0.013 |
| Proning | 0.0148 | 0.003 | 4.440 | 0.000 | 0.008 | 0.021 |
| pH | 31.5891 | 4.297 | 7.352 | 0.000 | 23.163 | 40.015 |
| PEEP | -0.2723 | 0.076 | -3.570 | 0.000 | -0.422 | -0.123 |
| P/F-ratio | 0.0221 | 0.004 | 5.281 | 0.000 | 0.014 | 0.030 |
| Is male | -1.9134 | 0.510 | -3.750 | 0.000 | -2.914 | -0.913 |
| Hematocrit | 23.8608 | 4.917 | 4.852 | 0.000 | 14.218 | 33.504 |
| Driving pressure | -0.2906 | 0.059 | -4.961 | 0.000 | -0.405 | -0.176 |
| C reactive protein | -0.0060 | 0.002 | -2.565 | 0.010 | -0.011 | -0.001 |
| Sodium | -0.0324 | 0.013 | -2.446 | 0.015 | -0.058 | -0.006 |
| Has renal comorb yes | -3.0113 | 1.253 | -2.404 | 0.016 | -5.468 | -0.554 |
| Ventilatory ratio | 1.1895 | 0.511 | 2.326 | 0.020 | 0.187 | 2.192 |
| Leukocytes | -0.0983 | 0.045 | -2.179 | 0.029 | -0.187 | -0.010 |
| Med steroids | 0.0065 | 0.003 | 2.075 | 0.038 | 0.000 | 0.013 |
| Heart rate | -0.0308 | 0.015 | -2.052 | 0.040 | -0.060 | -0.001 |
| Temperature | -0.5017 | 0.262 | -1.916 | 0.055 | -1.015 | 0.012 |
| Bicarbonate | -0.1076 | 0.057 | -1.879 | 0.060 | -0.220 | 0.005 |
| Glucose | -0.1972 | 0.106 | -1.860 | 0.063 | -0.405 | 0.011 |
| D dimer | -0.0002 | 0.000 | -1.197 | 0.232 | -0.001 | 0.000 |
| Tidal volume per kg | -0.2784 | 0.238 | -1.170 | 0.242 | -0.745 | 0.188 |
| Has lung comorb yes | -0.7885 | 0.738 | -1.068 | 0.286 | -2.236 | 0.659 |
| Potassium | 0.4648 | 0.440 | 1.057 | 0.291 | -0.398 | 1.327 |
| Blood pressure mean | 0.0267 | 0.026 | 1.010 | 0.313 | -0.025 | 0.079 |
| Has cancer comorb yes | -1.5350 | 1.605 | -0.957 | 0.339 | -4.682 | 1.612 |
| Has aki comorb yes | 0.7091 | 0.790 | 0.897 | 0.370 | -0.841 | 2.259 |
| Fluid balance | 0.0000 | 0.000 | 0.743 | 0.458 | 0.000 | 0.000 |
| Creatinine | -0.0021 | 0.003 | -0.732 | 0.464 | -0.008 | 0.003 |
| Respiratory rate measured | -0.0417 | 0.063 | -0.659 | 0.510 | -0.166 | 0.082 |
| Days | -0.0462 | 0.073 | -0.636 | 0.525 | -0.189 | 0.096 |
| Has imm insuf comorb yes | -0.4023 | 0.906 | -0.444 | 0.657 | -2.179 | 1.374 |
| Has diabetes comorb yes | 0.2497 | 0.586 | 0.426 | 0.670 | -0.899 | 1.398 |
| Bilirubin total | -0.0080 | 0.021 | -0.377 | 0.706 | -0.050 | 0.034 |
| Ureum | -0.0102 | 0.037 | -0.273 | 0.785 | -0.083 | 0.063 |
| Ventilation mode | -0.1501 | 0.608 | -0.247 | 0.805 | -1.342 | 1.042 |
| Albumin | 0.0112 | 0.056 | 0.199 | 0.843 | -0.099 | 0.122 |
| Body mass index | 0.0069 | 0.046 | 0.150 | 0.881 | -0.083 | 0.097 |
| Med vasopressors | 0.0007 | 0.006 | 0.123 | 0.902 | -0.010 | 0.012 |

## **Additional Table 4.** Predictor correlations

| **Predictor 1** | **Predictor 2** | **Pearson Coefficient** |
| --- | --- | --- |
| FiO2 | P/F-Ratio | 0.79 |
| Driving pressure | Lung compliance static | 0.74 |
| Creatinine | Ureum | 0.70 |
| Driving pressure | Plateau pressure | 0.68 |
| PCO2 (art.) | pH | 0.66 |
| PEEP | Plateau pressure | 0.64 |
| PCO2 (art.) | Ventilatory ratio | 0.58 |
| Lung compliance static | Plateau pressure | 0.57 |
| Respiratory rate | Ventilatory ratio | 0.55 |
| P/F-Ratio | PO2 (Art.) | 0.52 |

*Predictors are dropped by an intensive care physician in case of a Pearson correlation coefficient larger than 0.5*
